# Supplementary material for: Cramming versus threading of long amphiphilic oligomers into a polyaromatic capsule
Source: Nat Commun. 2018 Oct 12;9:4227. doi: 10.1038/s41467-018-06458-w (PMC6185989; doi:10.1038/s41467-018-06458-w)
Supplement: Supplementary file 1 — Supplementary Information [file 41467_2018_6458_MOESM1_ESM.pdf]

Supplementary Information

Cramming versus threading of long amphiphilic oligomers into a polyaromatic capsule

Yamashina et al.

Supplementary Figures

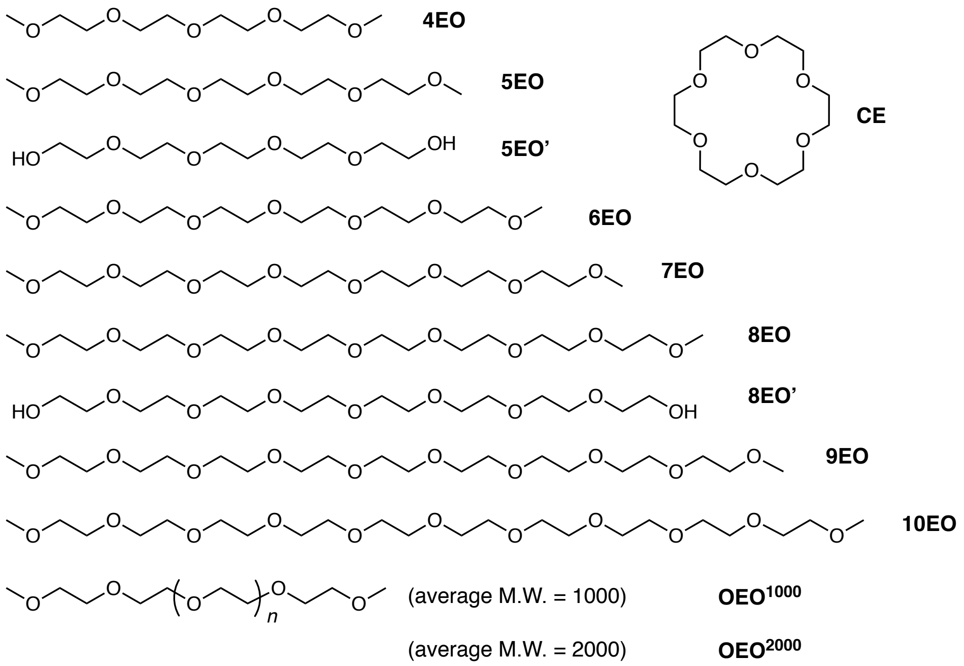

Supplementary Fig. 1 Chemical structures of oligo(ethylene oxide)s and the derivatives.

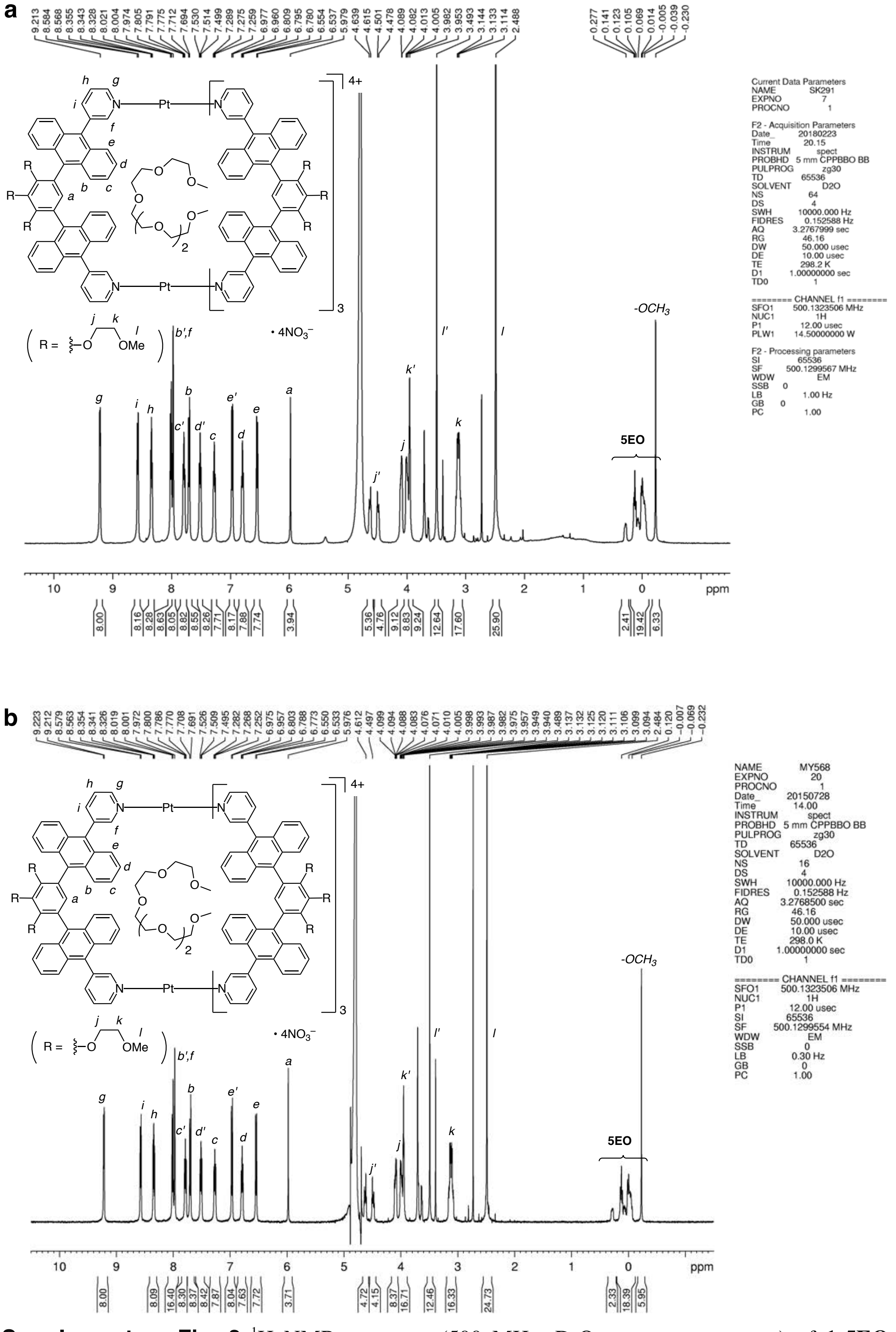

Supplementary Fig. 2 ^{1}H NMR spectrum (500 MHz, D_2O, room temperature) of **1•5EO** (prepared at (a) room temperature and (b) 60 ºC).

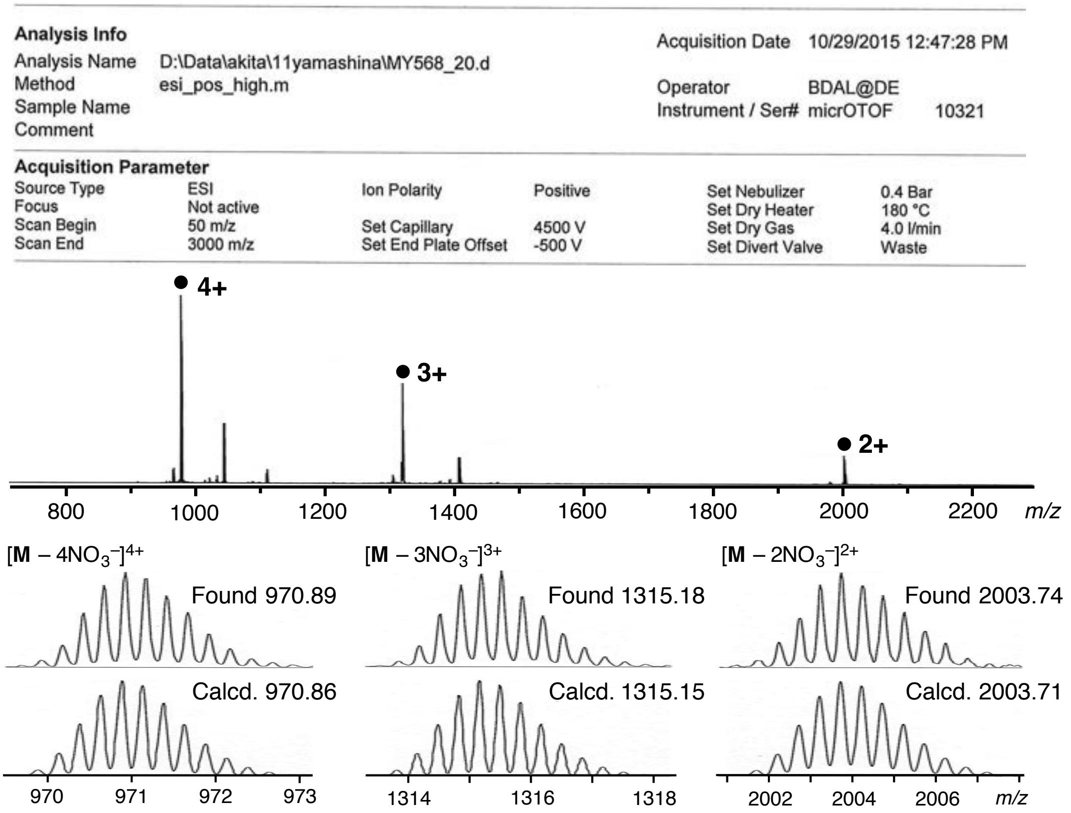

Supplementary Fig. 3 ESI-TOF MS spectrum (H_2O) of **1•5EO**.

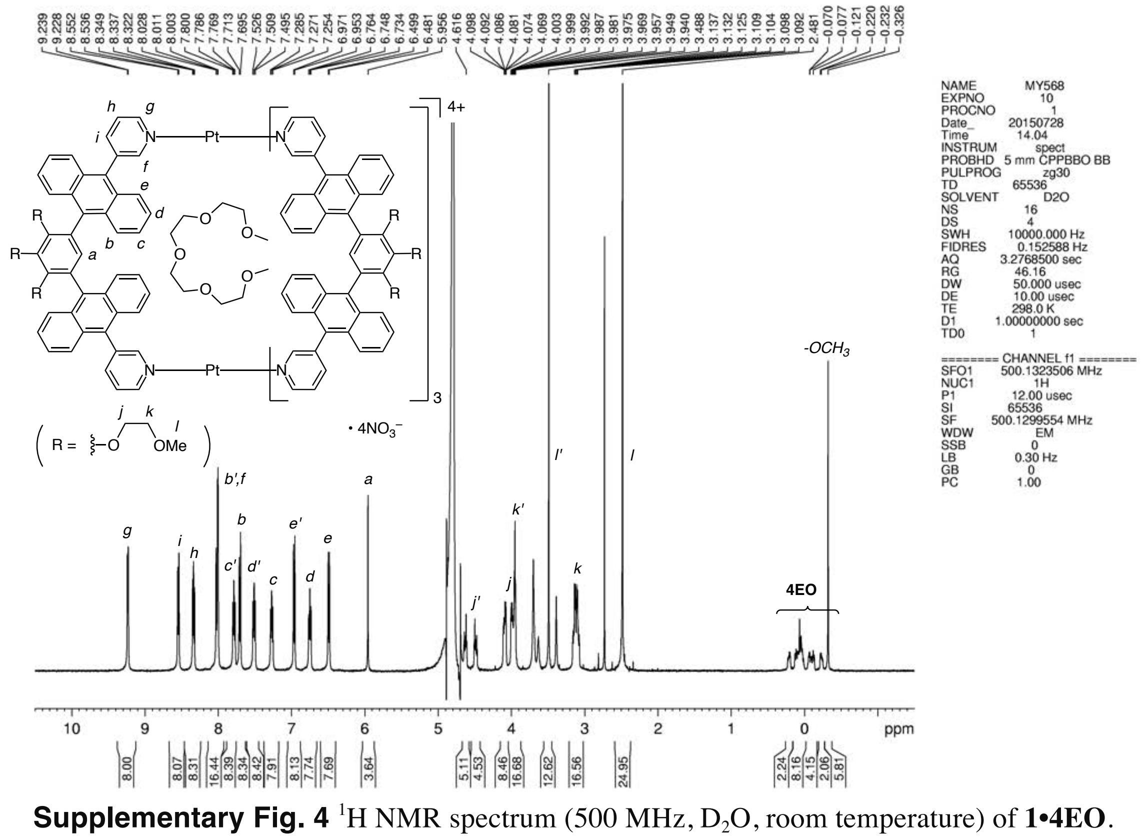

Supplementary Fig. 4 ^{1}H NMR spectrum (500 MHz, D_2O, room temperature) of **1•4EO**.

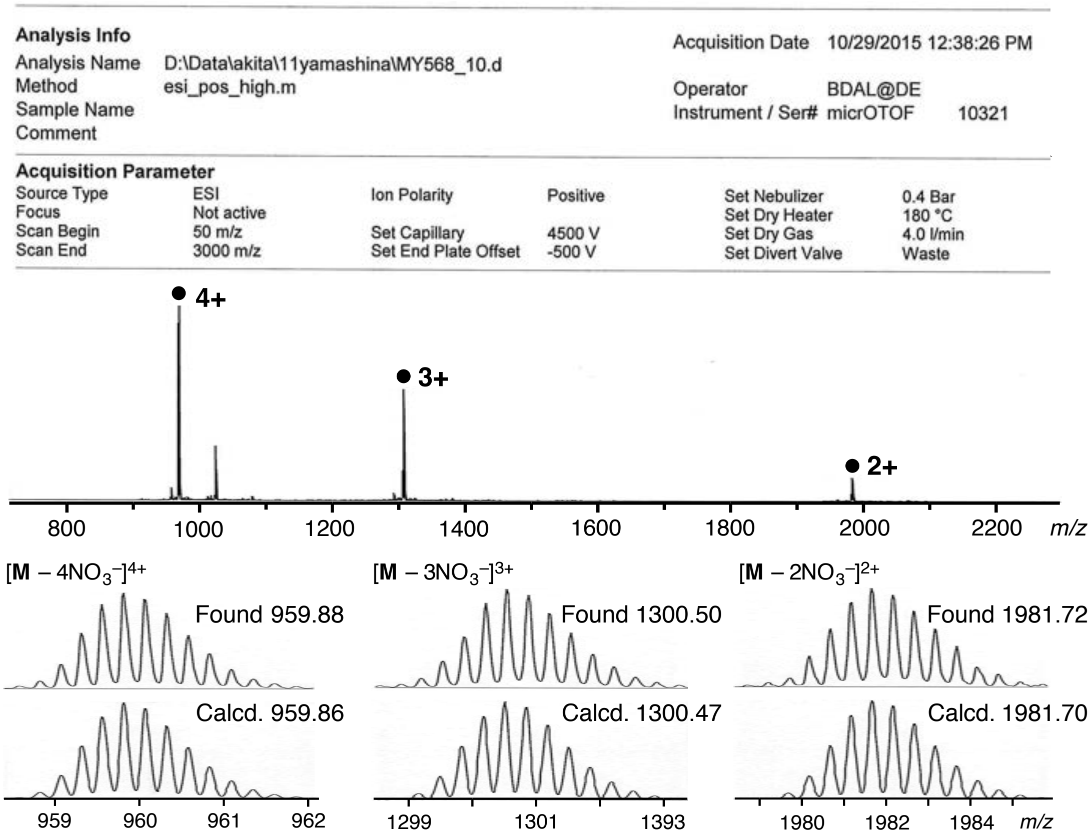

Supplementary Fig. 5 ESI-TOF MS spectrum (H_2O) of **1•4EO**.

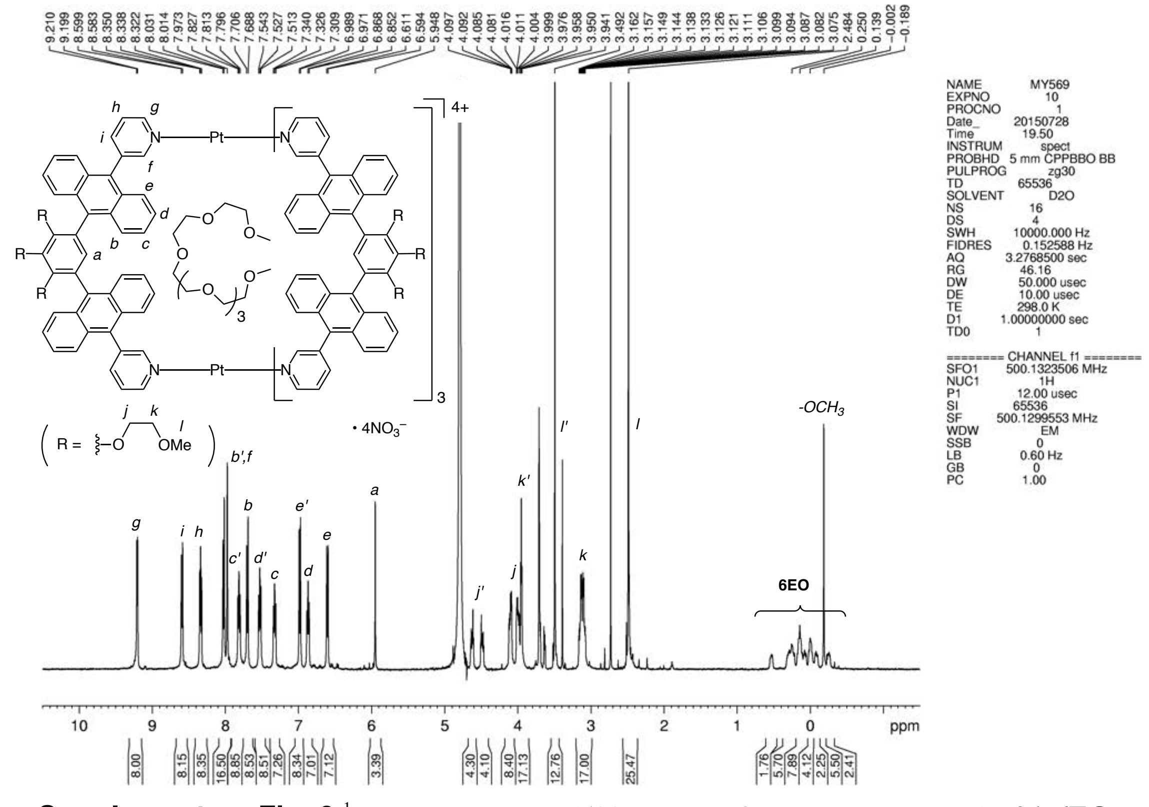

Supplementary Fig. 6 ^{1}H NMR spectrum (500 MHz, D_2O, room temperature) of **1•6EO**.

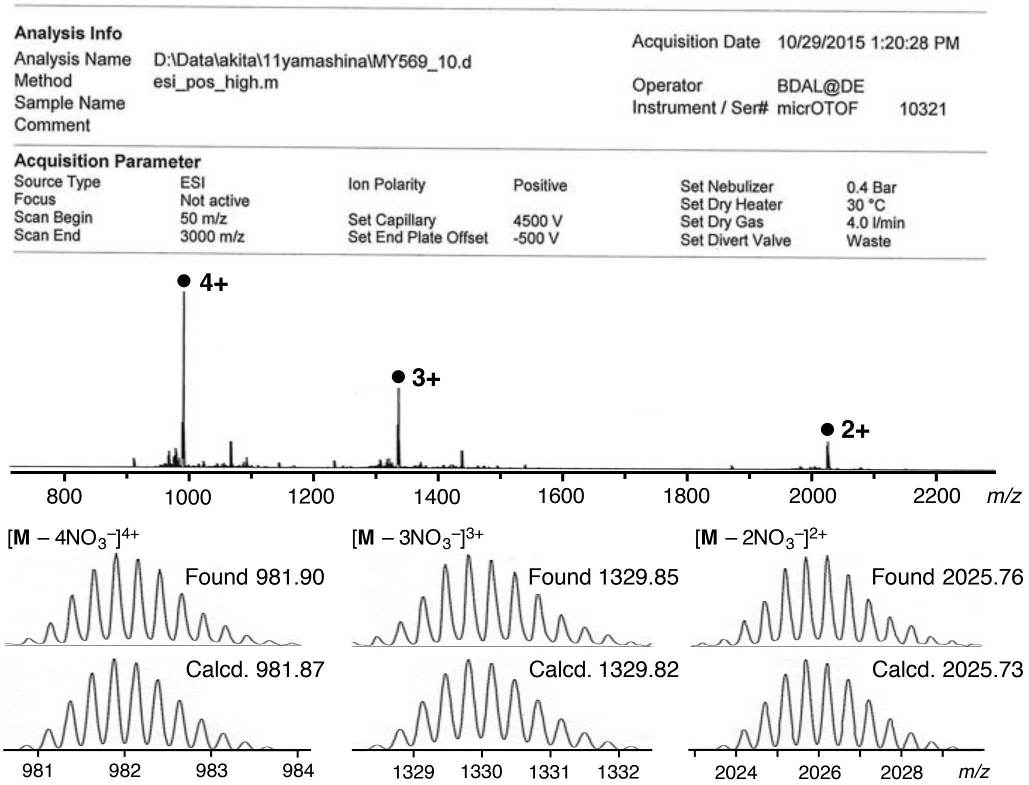

Supplementary Fig. 7 ESI-TOF MS spectrum (H_2O) of **1•6EO**.

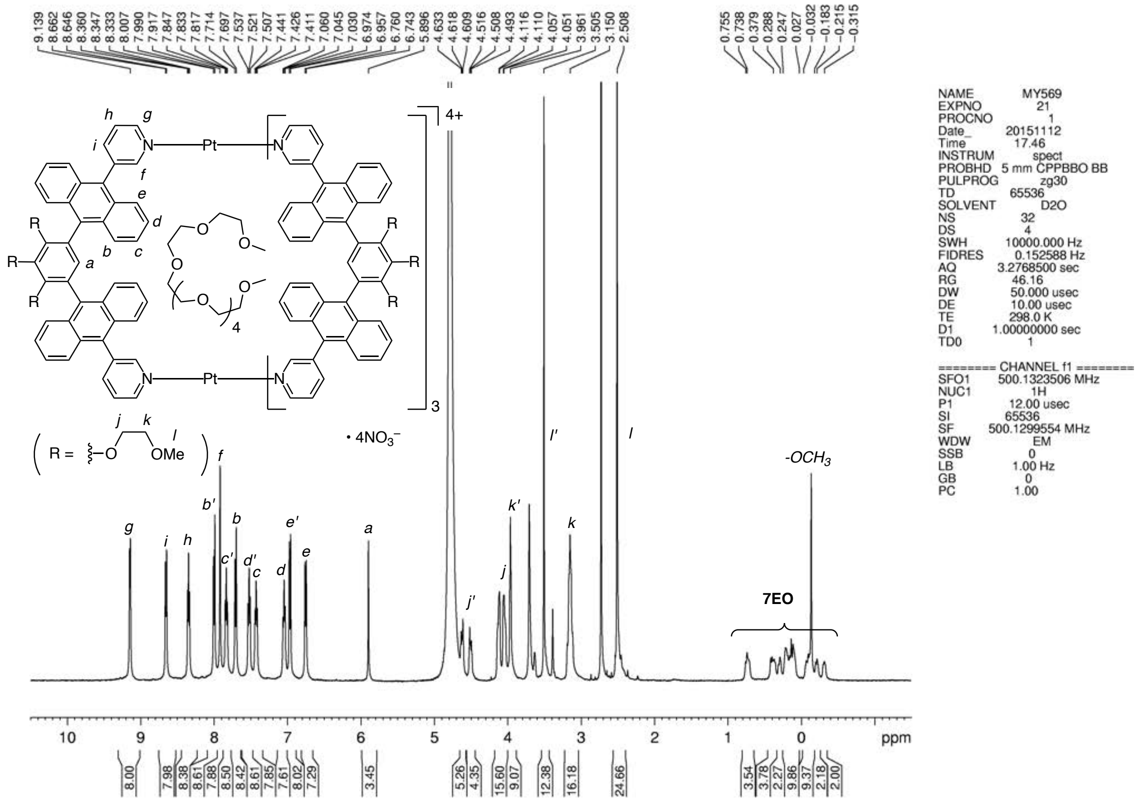

Supplementary Fig. 8 ^1H NMR spectrum (500 MHz, D_2O, room temperature) of **1•7EO**.

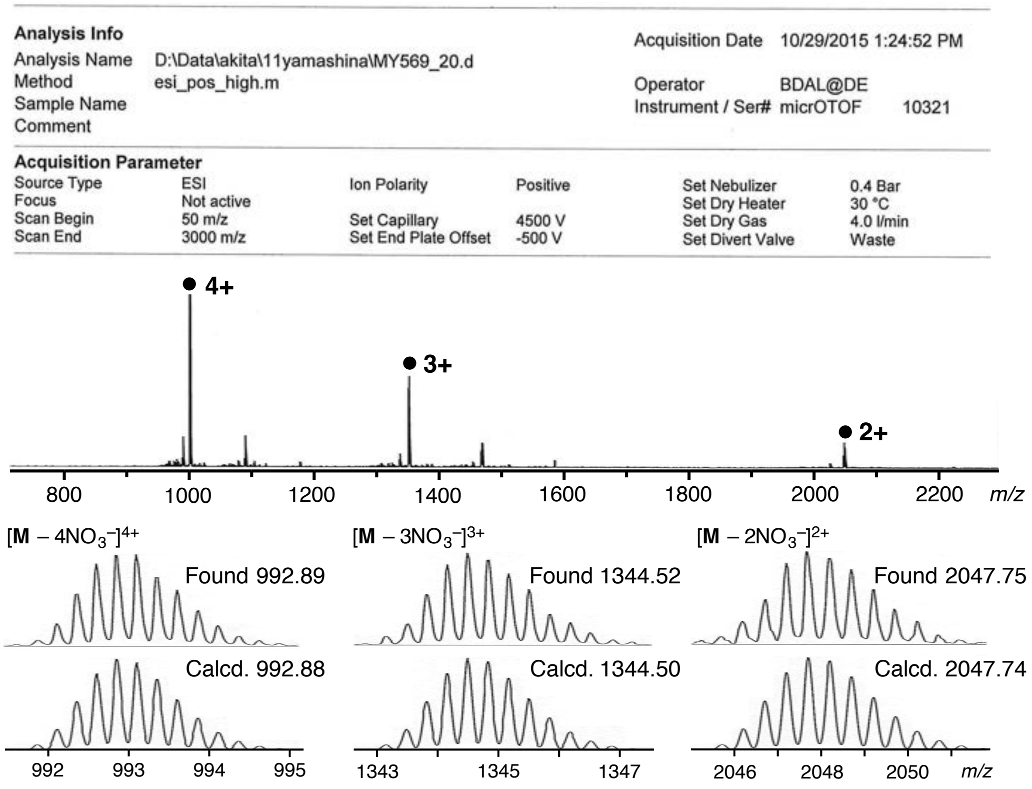

Supplementary Fig. 9 ESI-TOF MS spectrum (H_2O) of **1•7EO**.

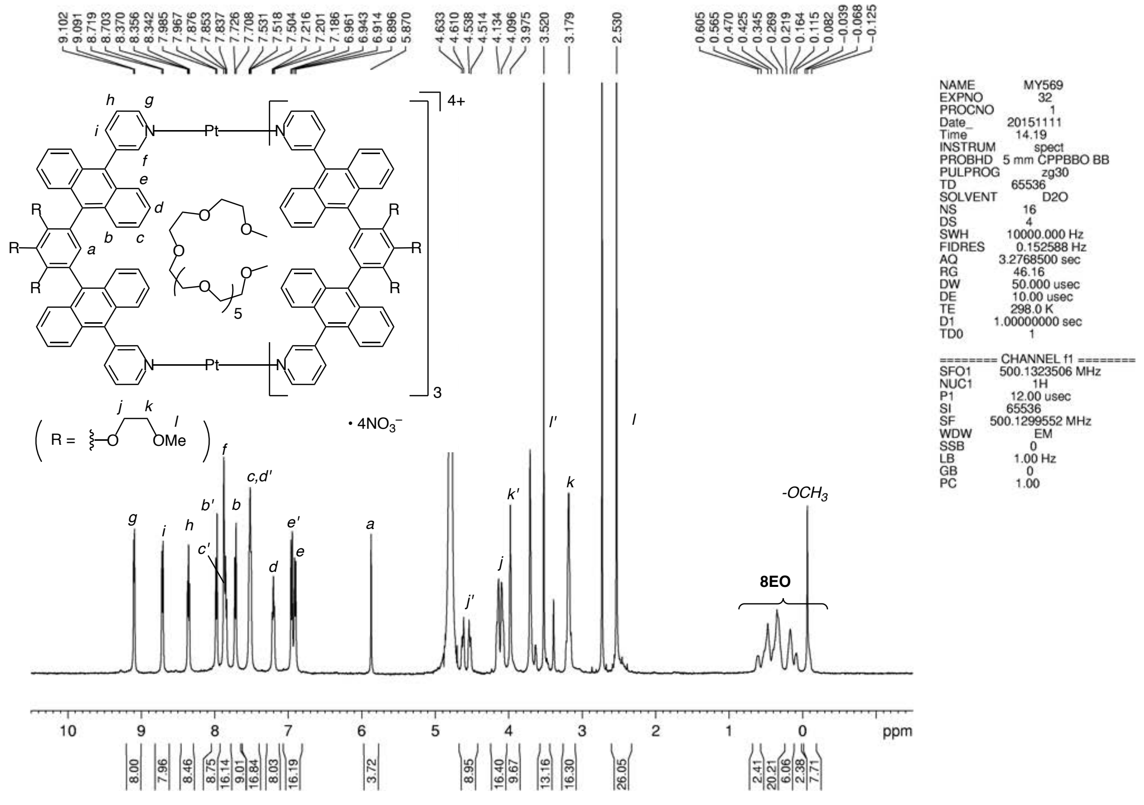

Supplementary Fig. 10 ^{1}H NMR spectrum (500 MHz, D_2O, room temperature) of **1•8EO**.

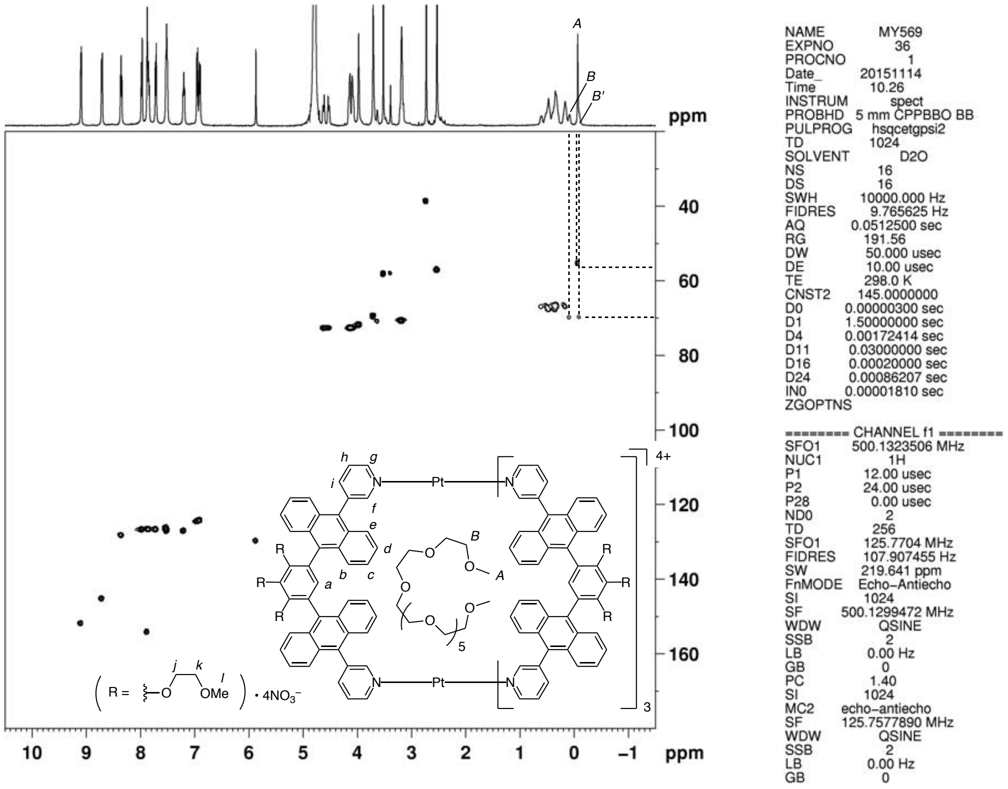

Supplementary Fig. 11 HSQC NMR spectrum (500 MHz, D_2O, room temperature) of **1•8EO**.

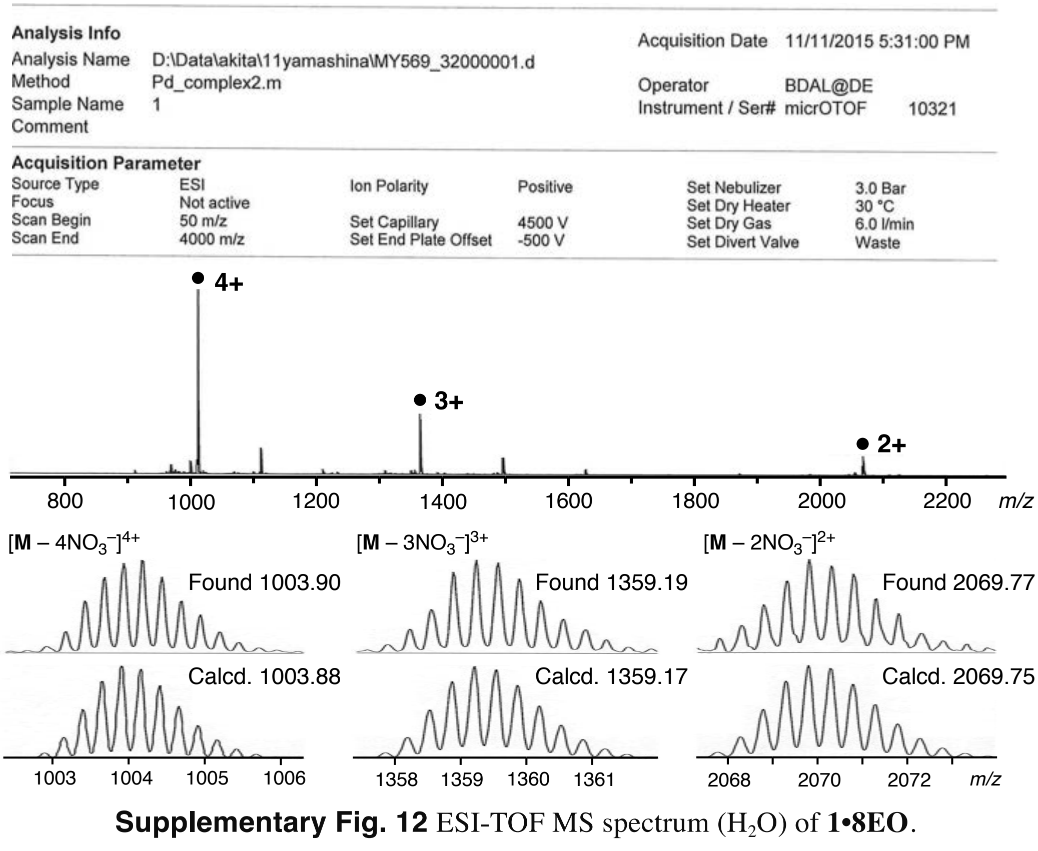

Supplementary Fig. 12 ESI-TOF MS spectrum (H_2O) of **1•8EO**.

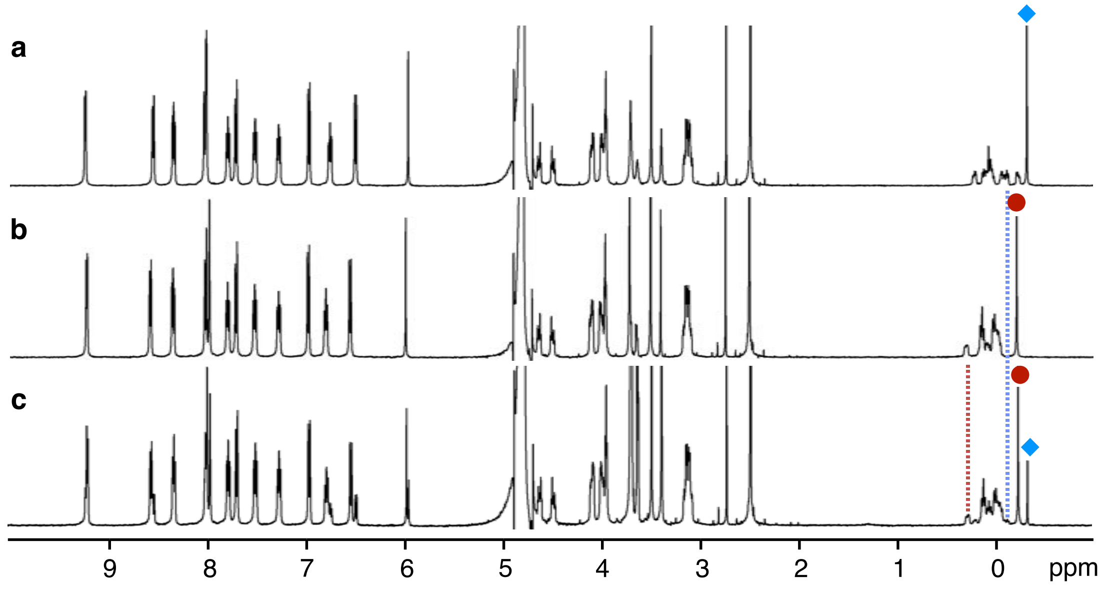

Supplementary Fig. 13 ^{1}H NMR spectra (500 MHz, D_2O, room temperature) of (a) **1•4EO** and (b) **1•5EO**, and (c) after mixing **4EO** and **5EO** with **1**.

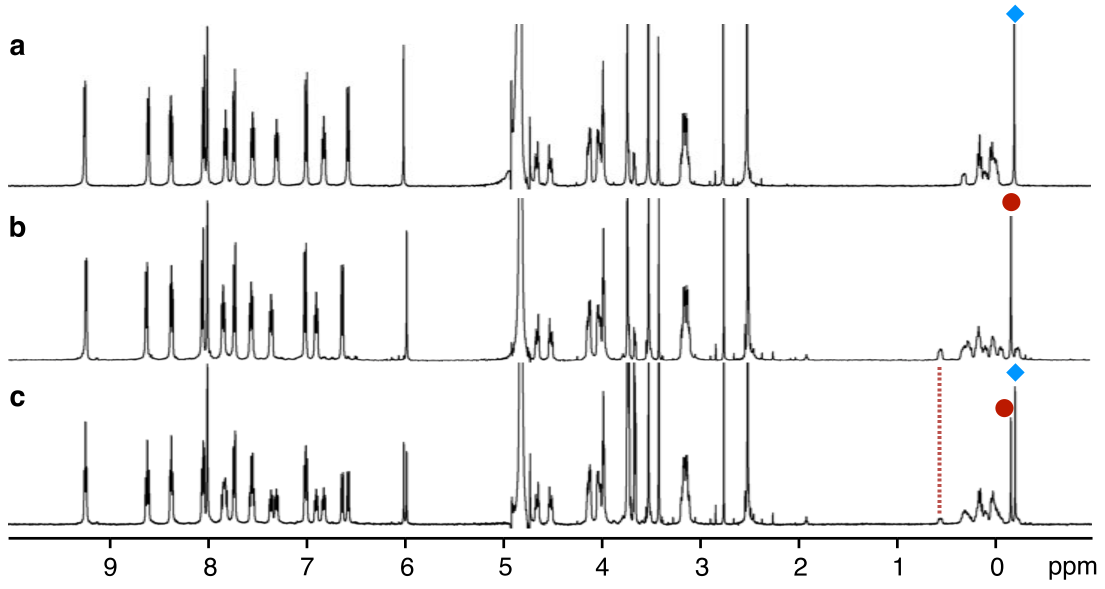

Supplementary Fig. 14 ^{1}H NMR spectra (500 MHz, D_2O, room temperature) of (a) **1•5EO** and (b) **1•6EO**, and (c) after mixing **5EO** and **6EO** with **1**.

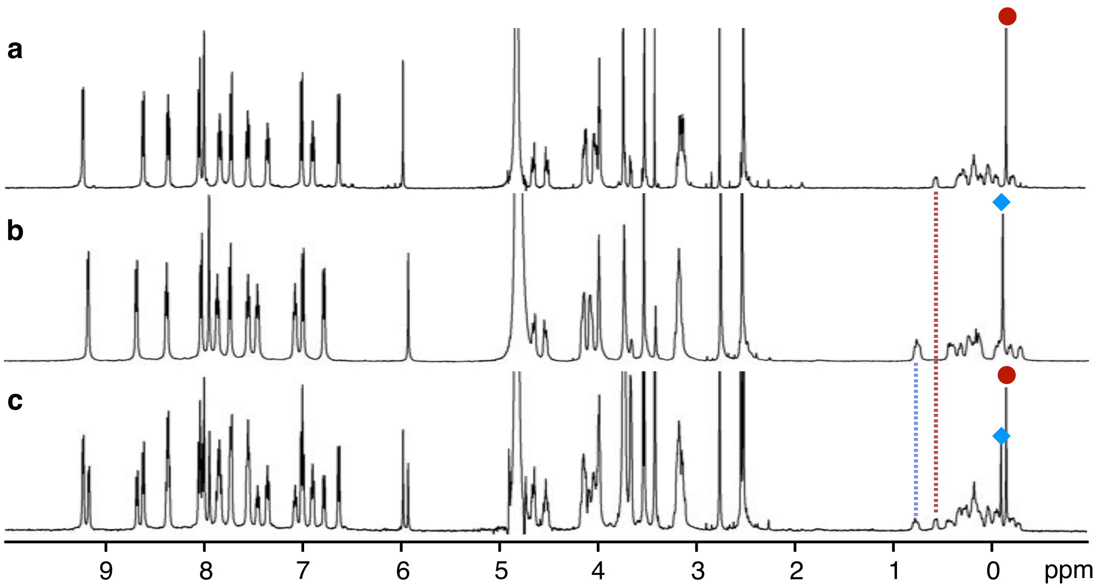

Supplementary Fig. 15 ^{1}H NMR spectra (500 MHz, D_2O, room temperature) of (a) **1•6EO** and (b) **1•7EO**, and (c) after mixing **6EO** and **7EO** with **1**.

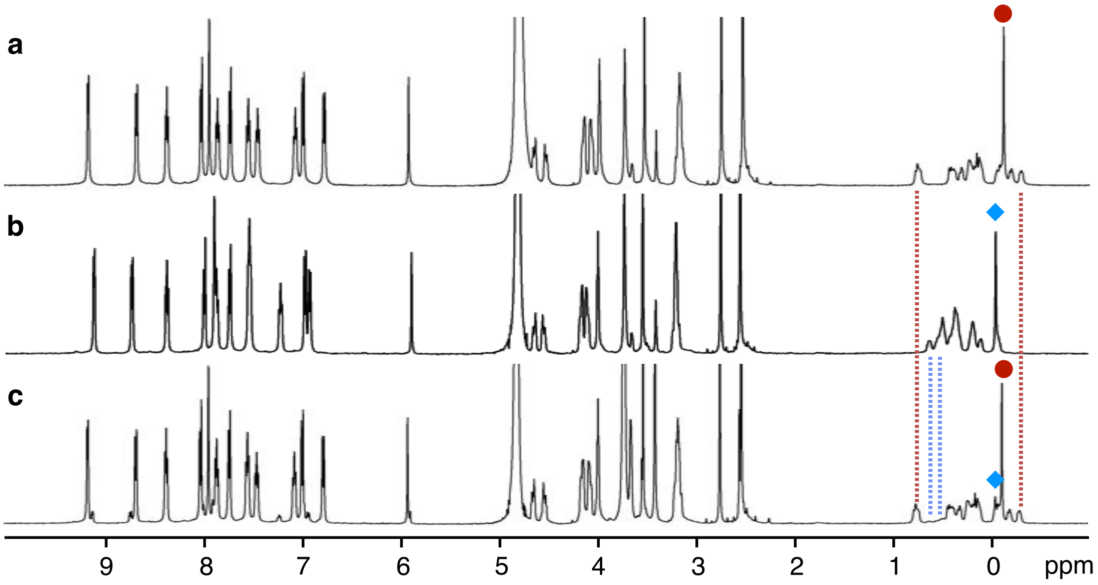

Supplementary Fig. 16 ^{1}H NMR spectra (500 MHz, D_2O, room temperature) of (a) **1•7EO** and (b) **1•8EO**, and (c) after mixing **7EO** and **8EO** with **1**.

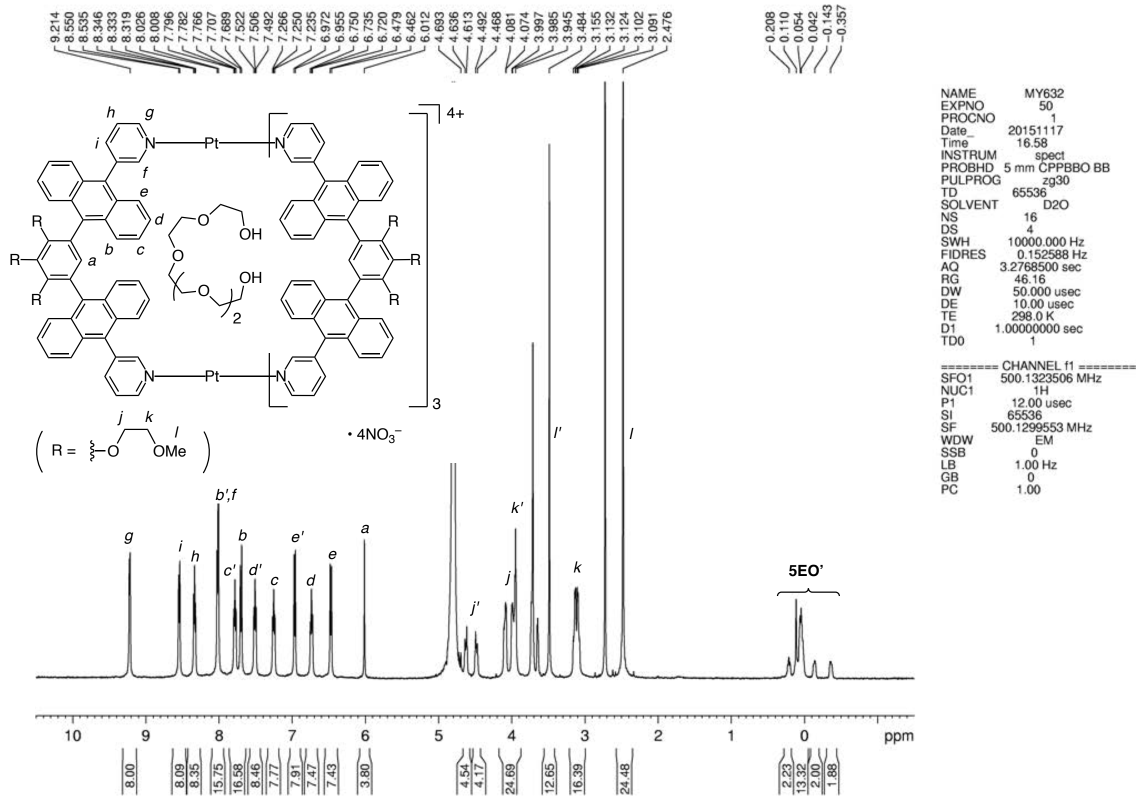

Supplementary Fig. 17 ^{1}H NMR spectrum (500 MHz, D_2O, room temperature) of **1•5EO'**.

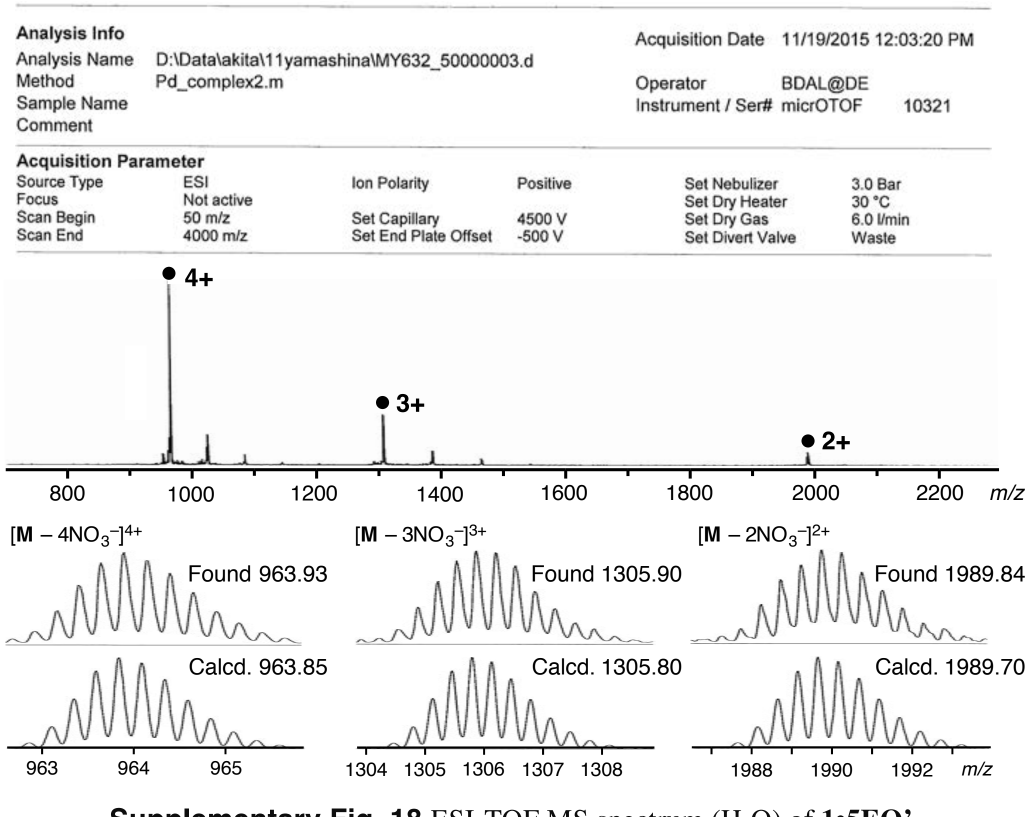

Supplementary Fig. 18 ESI-TOF MS spectrum (H_2O) of **1•5EO'**.

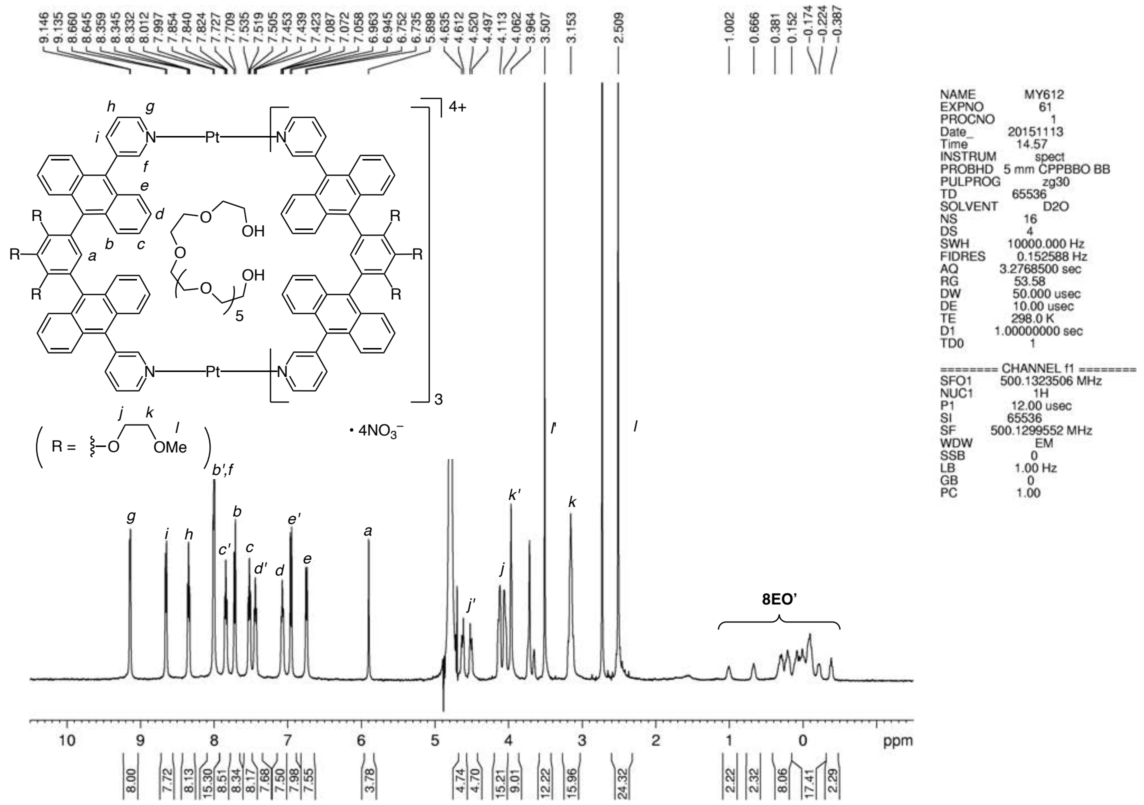

Supplementary Fig. 19 ^{1}H NMR spectrum (500 MHz, D_2O, room temperature) of **1•8EO'**.

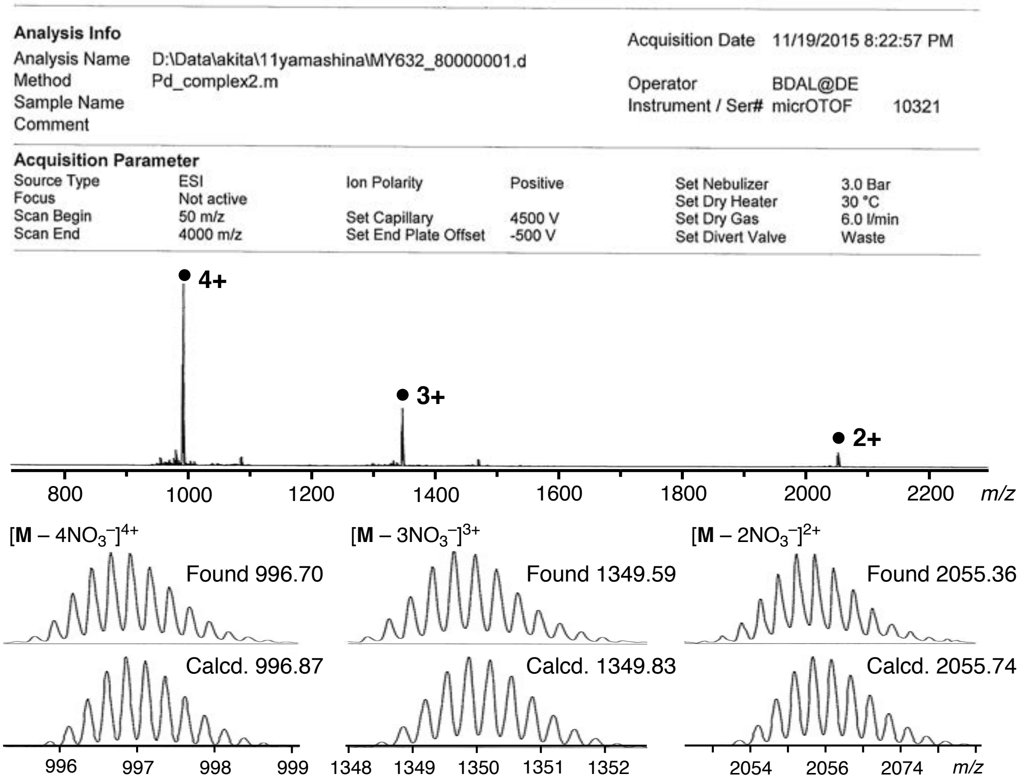

Supplementary Fig. 20 ESI-TOF MS spectrum (H_2O) of **1•8EO'**.

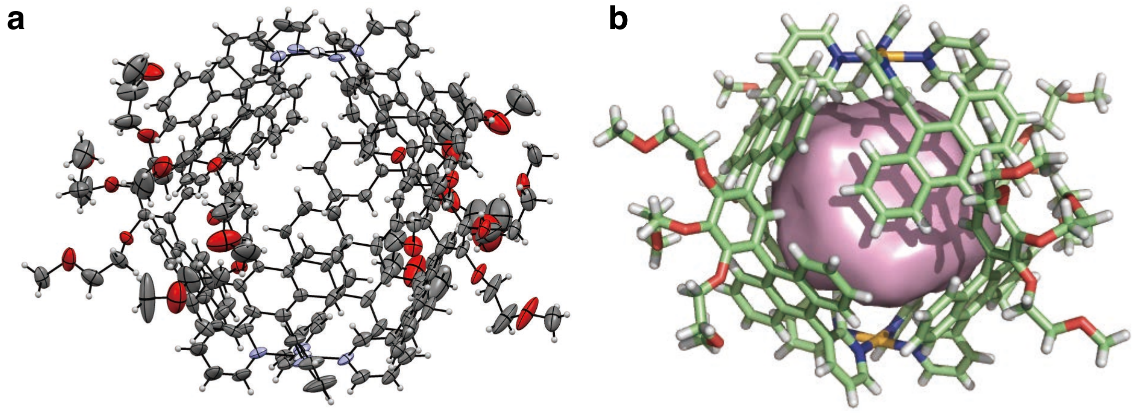

Supplementary Fig. 21 (a) The ORTEP drawing of the structure of **1**. The thermal ellipsoids are drawn at 30% probability. (b) The stick representation of **1** with calculated voido (solvent molecules and counternions are omitted for clarity).

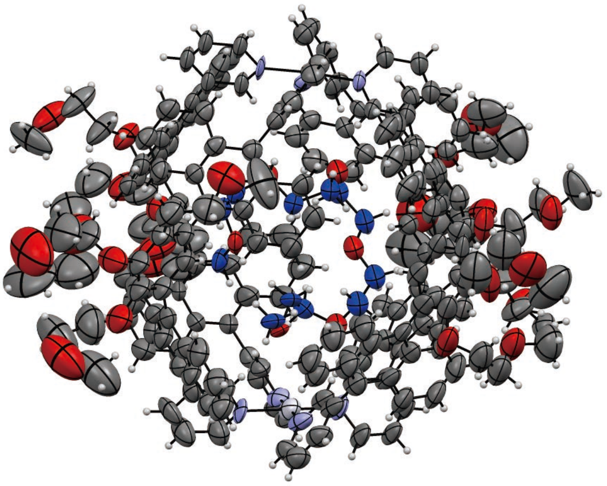

Supplementary Fig. 22 The ORTEP drawing of the structure of **1•5EO**. The thermal ellipsoids are drawn at 30% probability (solvent molecules and counternions are omitted for clarity).

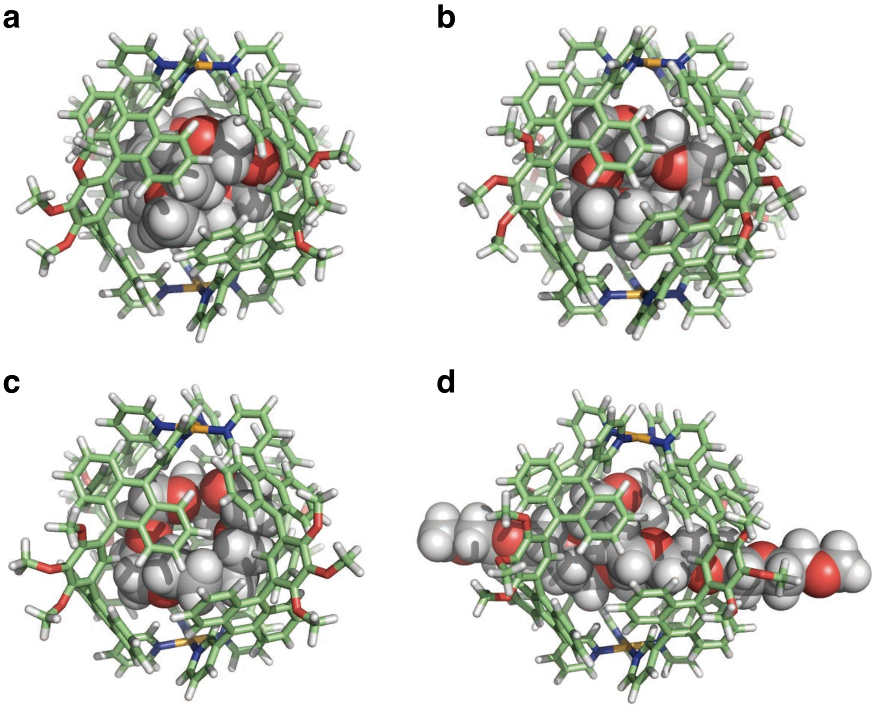

Supplementary Fig. 23 Optimized structures of (a) **1•6EO**, (b) **1•8EO**, (c) **1•CE**, and (d) **1•10EO** (R = -OCH_3).

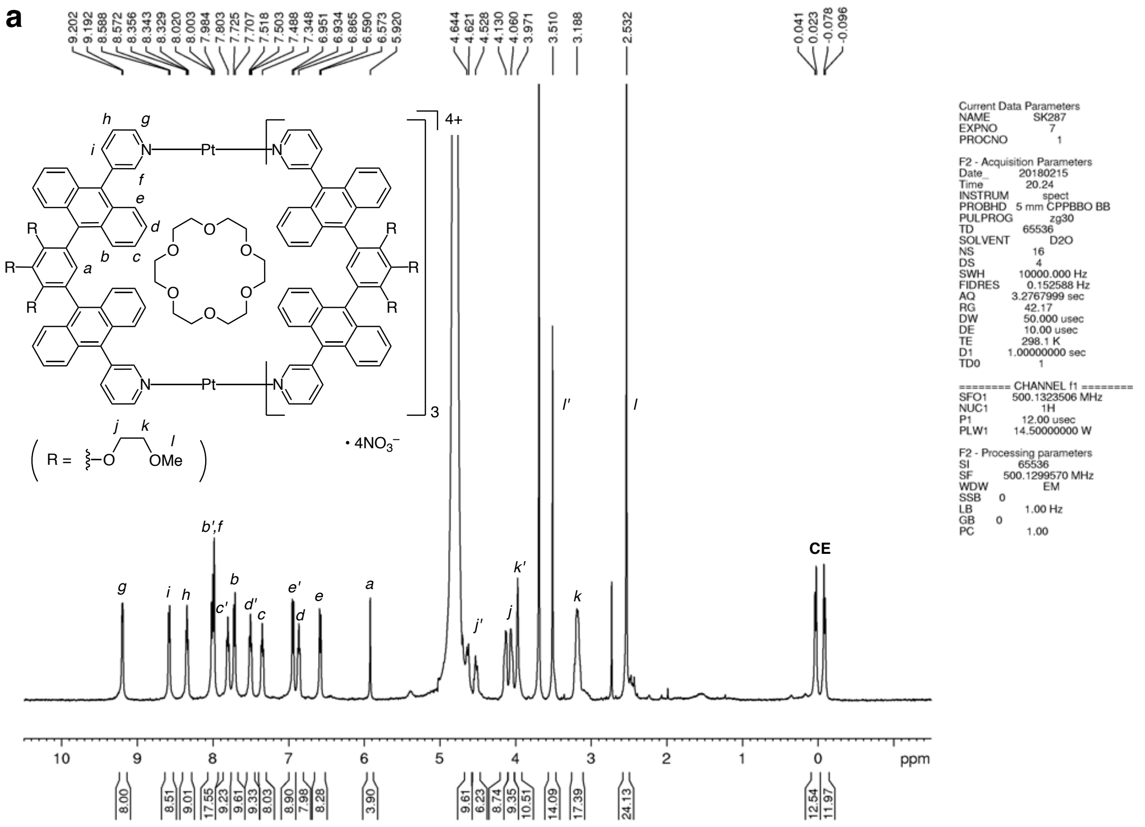

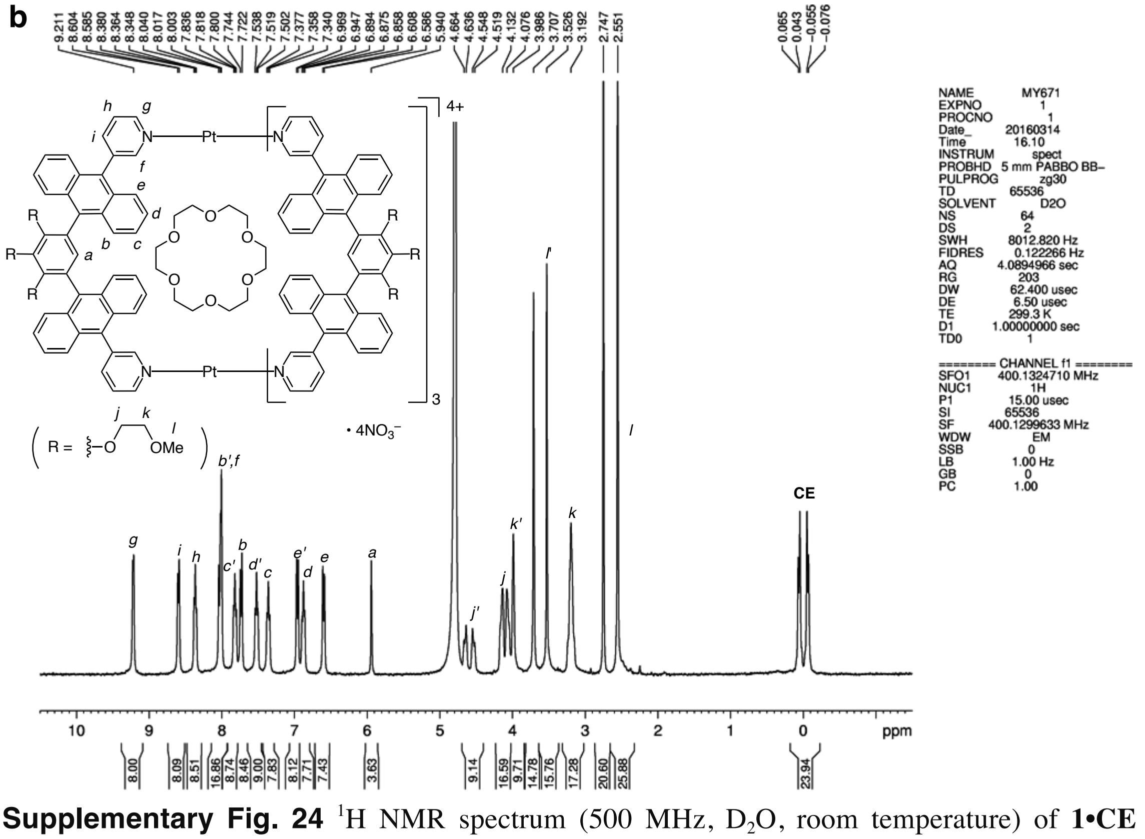

Supplementary Fig. 24 ^1H NMR spectrum (500 MHz, D_2O, room temperature) of **1•CE** (prepared at (a) room temperature and (b) 60 ºC).

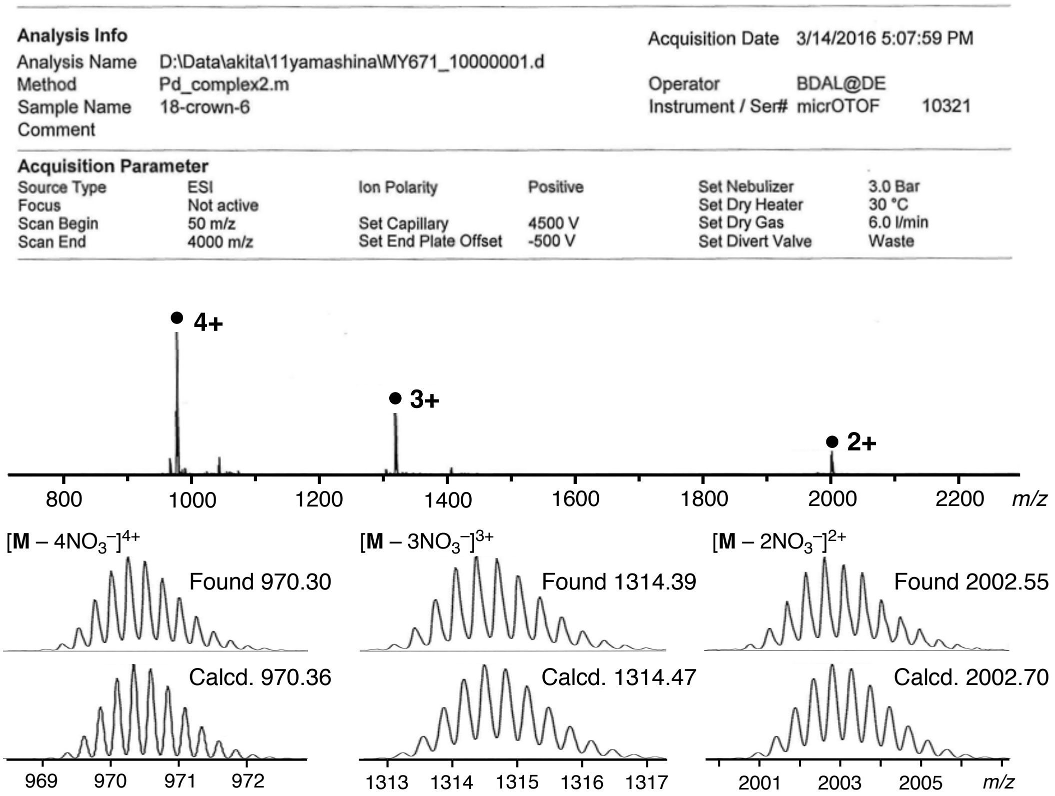

Supplementary Fig. 25 ESI-TOF MS spectrum (H_2O) of **1•CE**.

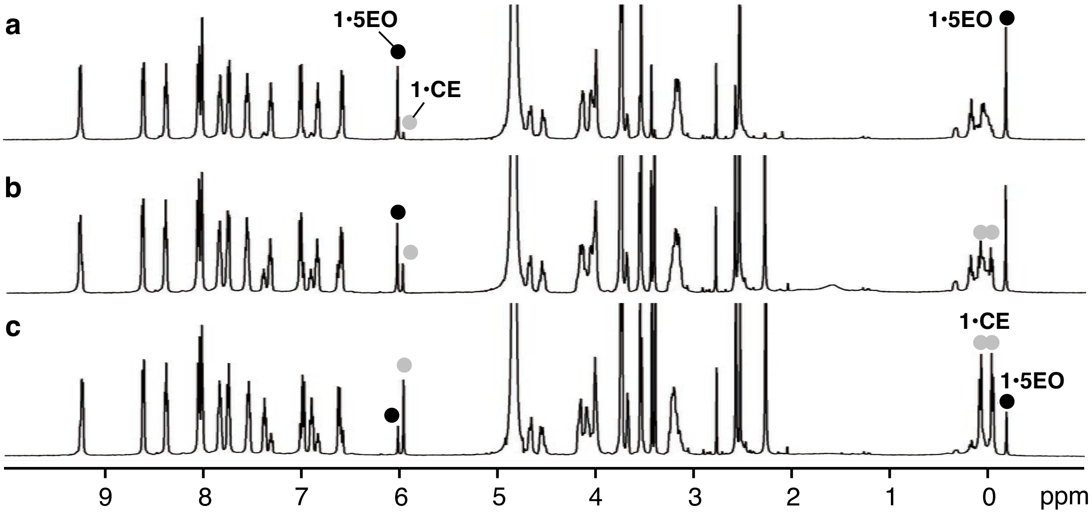

Supplementary Fig. 26 ^{1}H NMR spectra (500 MHz, D_2O, room temperature) of a mixture of **5EO**, **CE**, and **1** (1.0 equiv. each) after being stirred a) at room temperature for 30 min, b) at 60 ºC for 30 min, and c) at 60 ºC for 9 h.

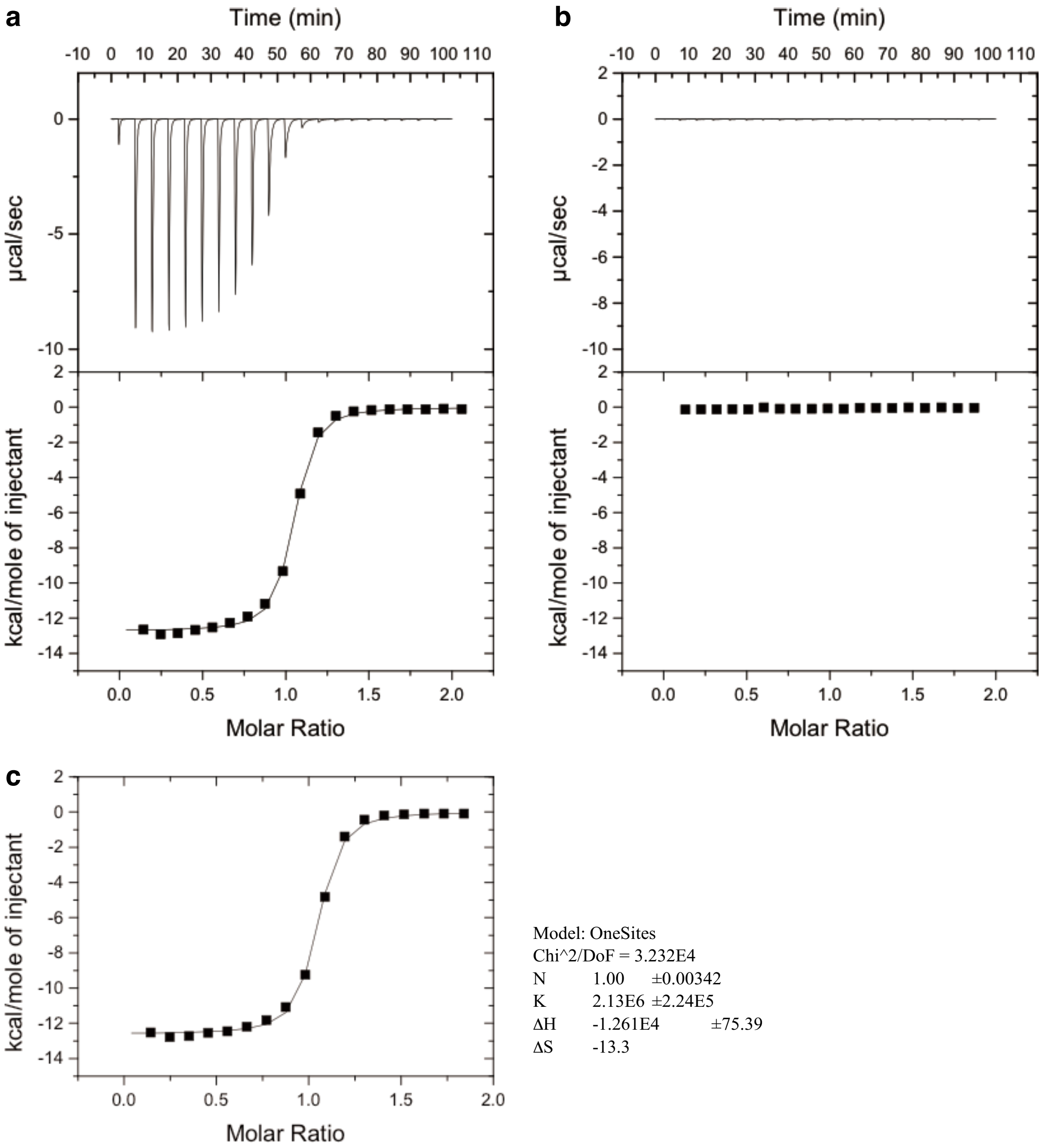

Supplementary Fig. 27 ITC titrations (H_2O, 25 ºC) of **5EO** (3.78 mM) in (a) the presence of **1** (0.13 mM) and (b) the absence of **1**. (c) The solid line represents the best-fitting curve obtained from the Onesites model.

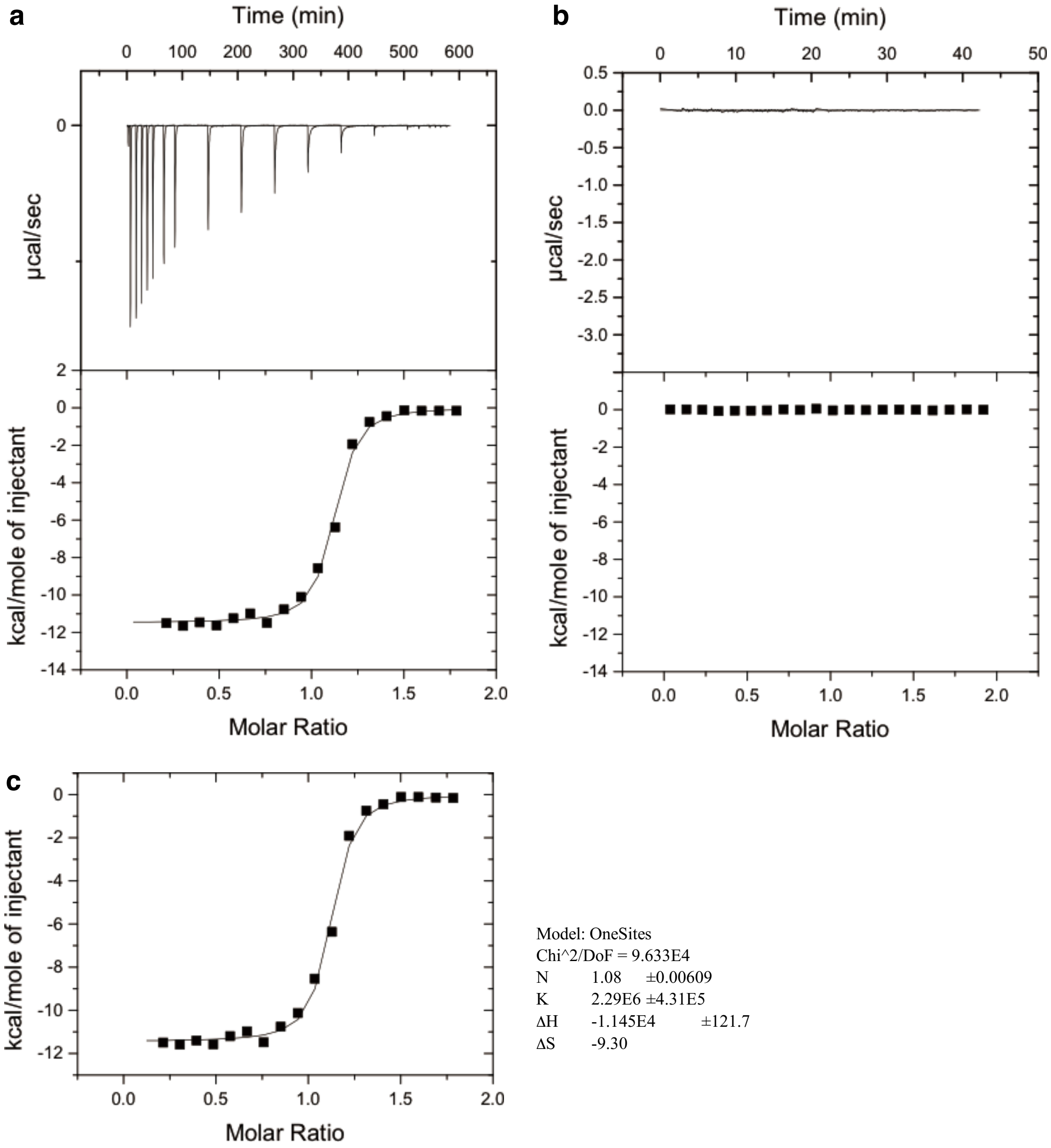

Supplementary Fig. 28 ITC titrations (H_2O, 25 ºC) of **CE** (3.30 mM) in (a) the presence of **1** (0.13 mM) and (b) the absence of **1**. (c) The solid line represents the best-fitting curve obtained from the Onesites model.

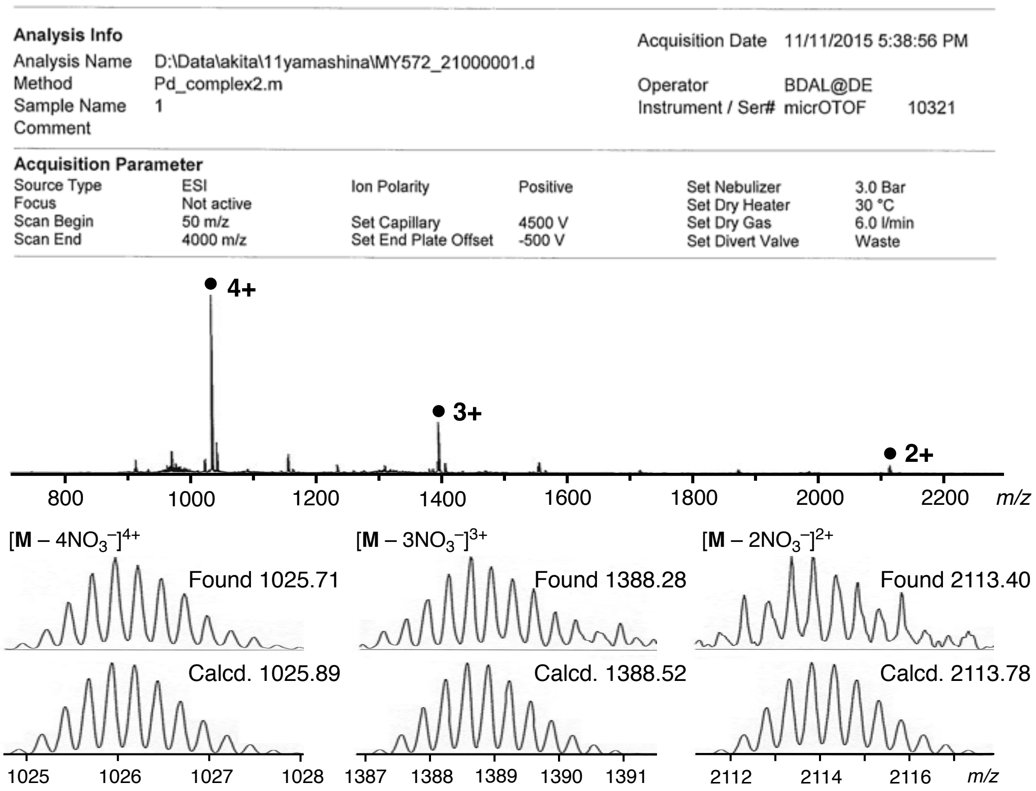

Supplementary Fig. 29 ESI-TOF MS spectrum (H_2O) of **1•10EO**.

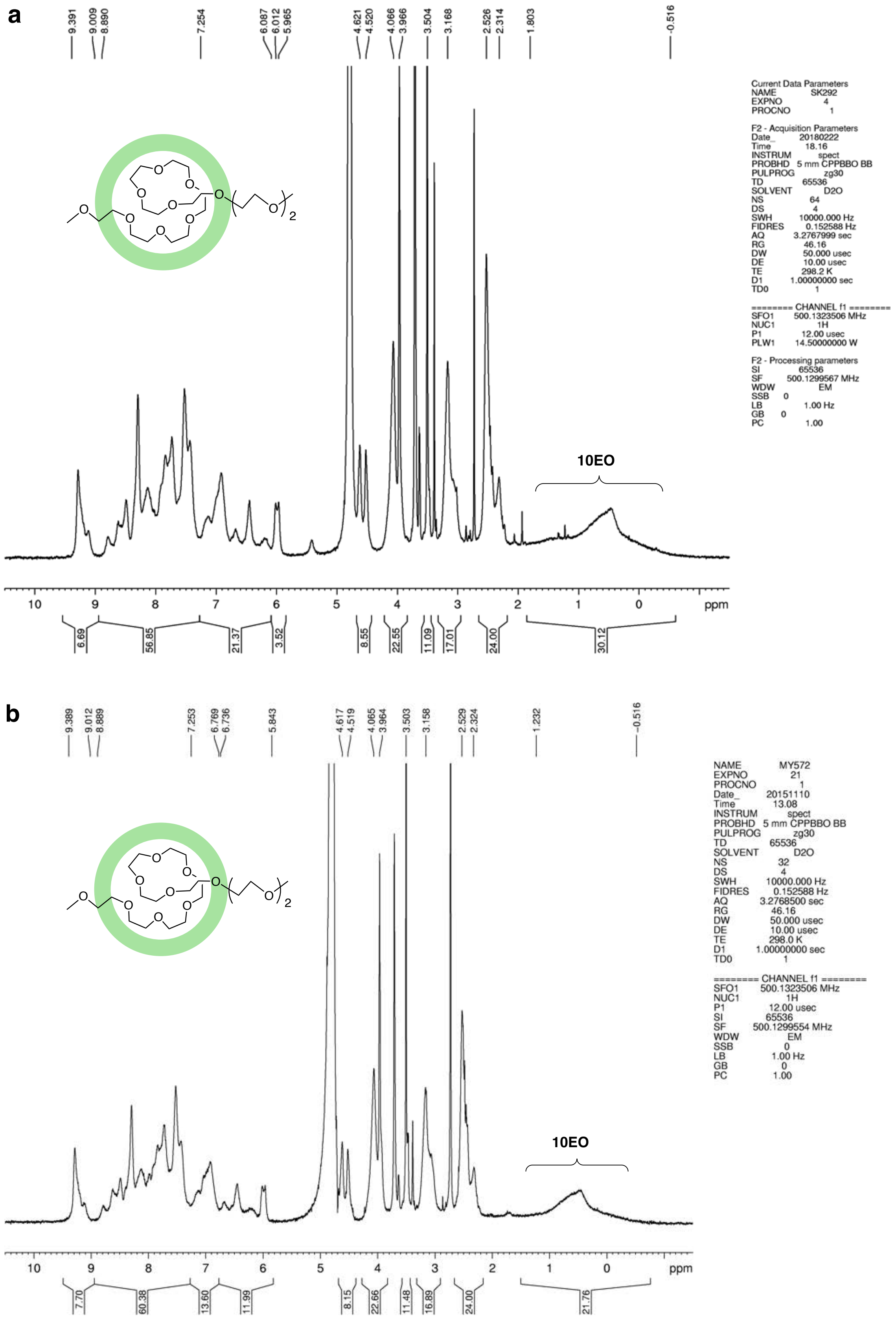

Supplementary Fig. 30 ^{1}H NMR spectrum (500 MHz, D_2O, room temperature) of **1•10EO** (prepared at (a) room temperature and (b) 60 ºC).

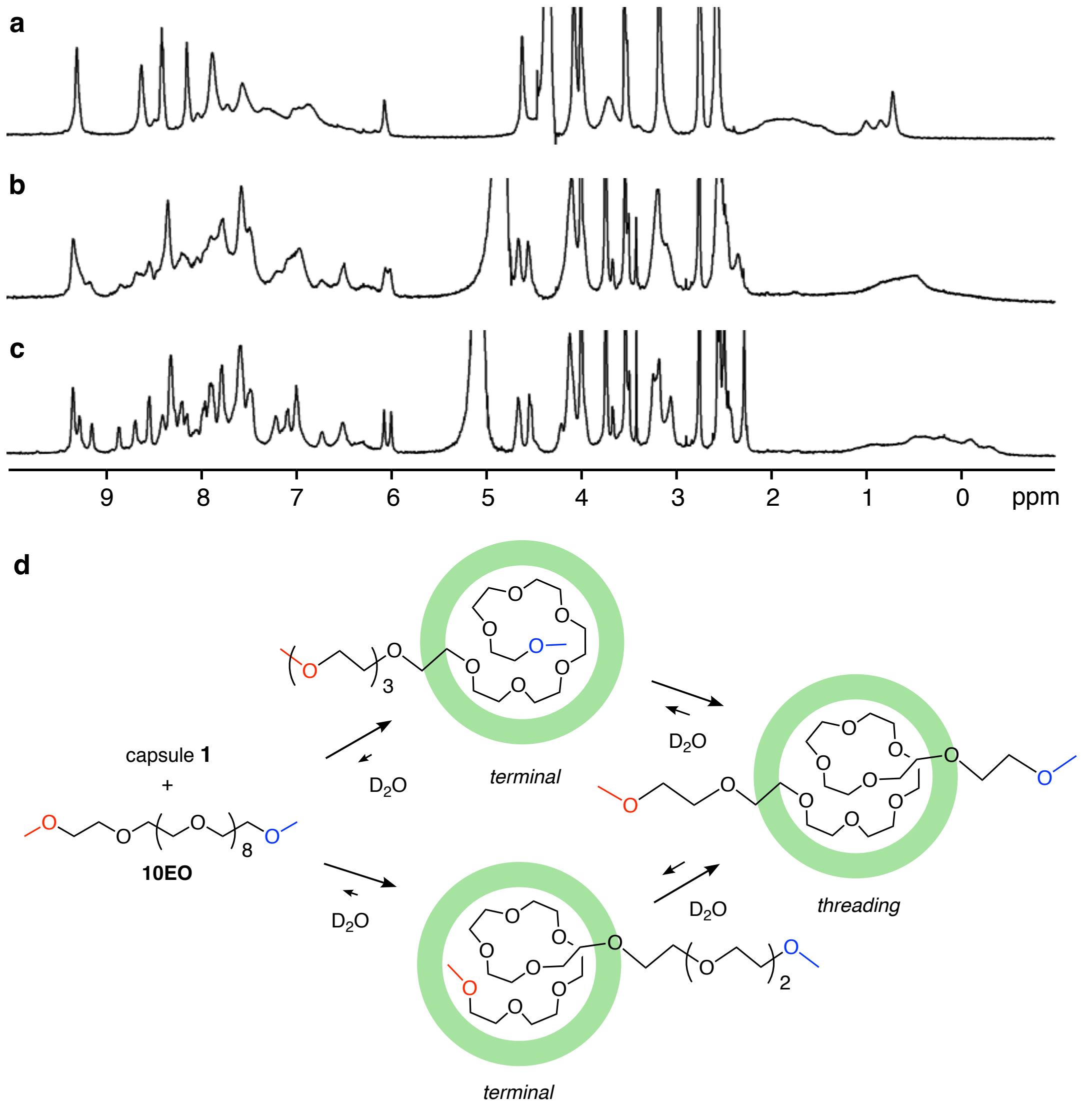

Supplementary Fig. 31 ^{1}H NMR spectra (500 MHz, D_2O) of **1•10EO** at (a) 75 ºC, (b) 25 ºC, and (c) 5 ºC. (d) Proposed dynamic behavior of **1•10EO** in water.

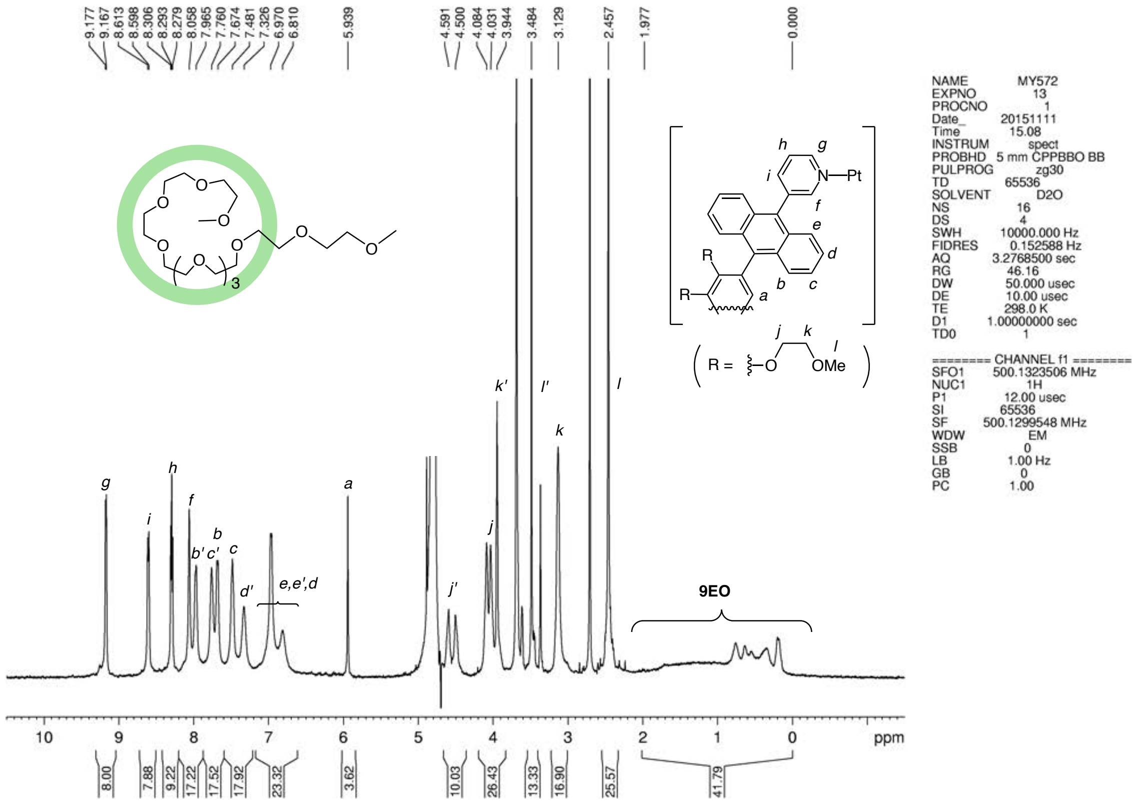

Supplementary Fig. 32 ^{1}H NMR spectrum (500 MHz, D_2O, room temperature) of **1•9EO**.

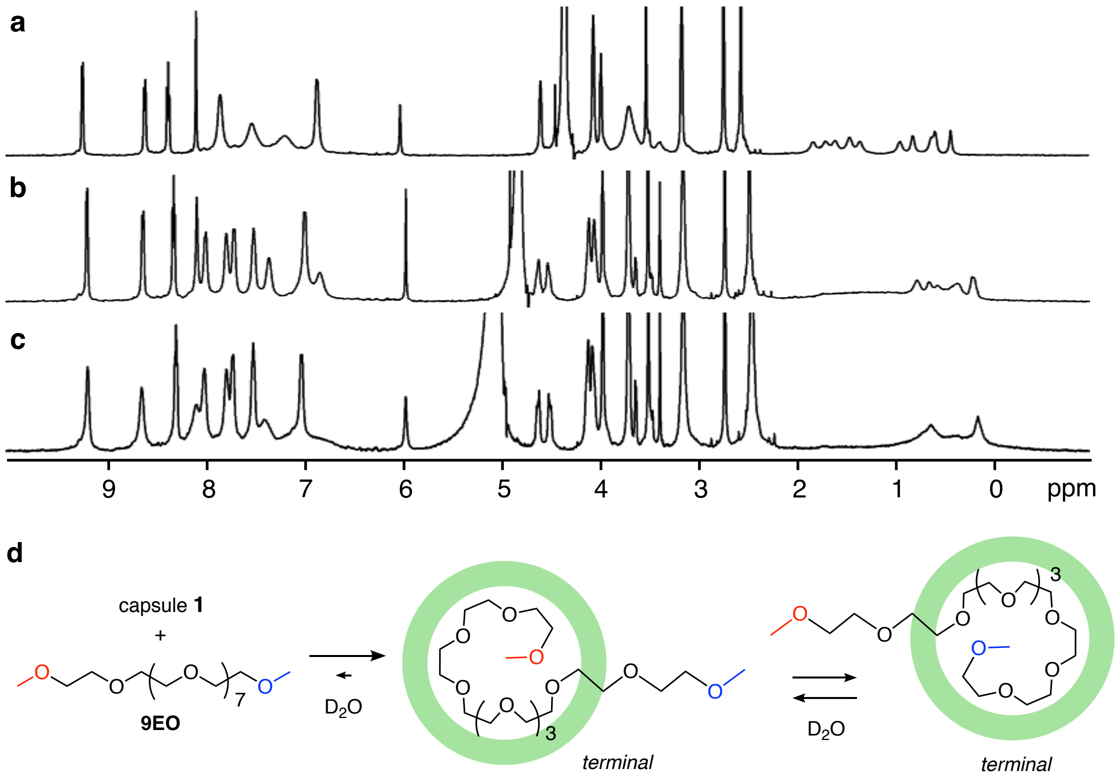

Supplementary Fig. 33 ^{1}H NMR spectra (500 MHz, D_2O) of **1•9EO** at (a) 75 ºC, (b) 25 ºC, and (c) 5 ºC. (d) Proposed dynamic behavior of **1•9EO** in water.

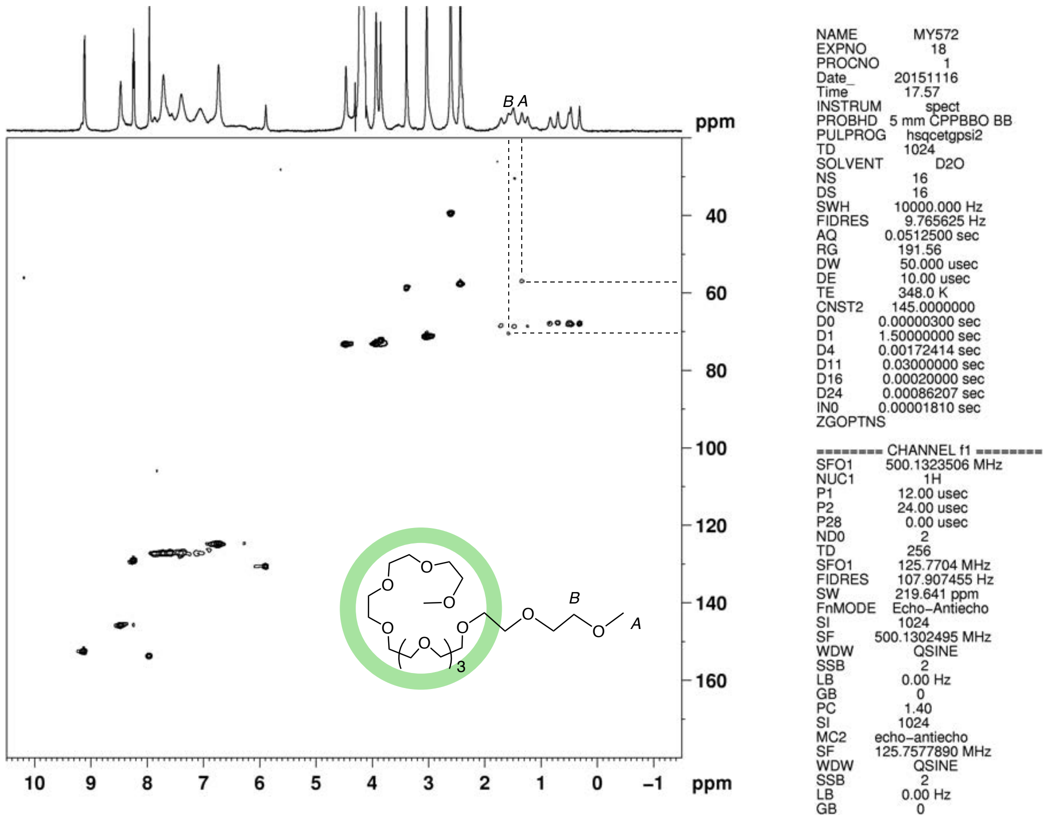

Supplementary Fig. 34 HSQC spectrum (500 MHz, D_2O, 75 ºC) of **1•9EO**.

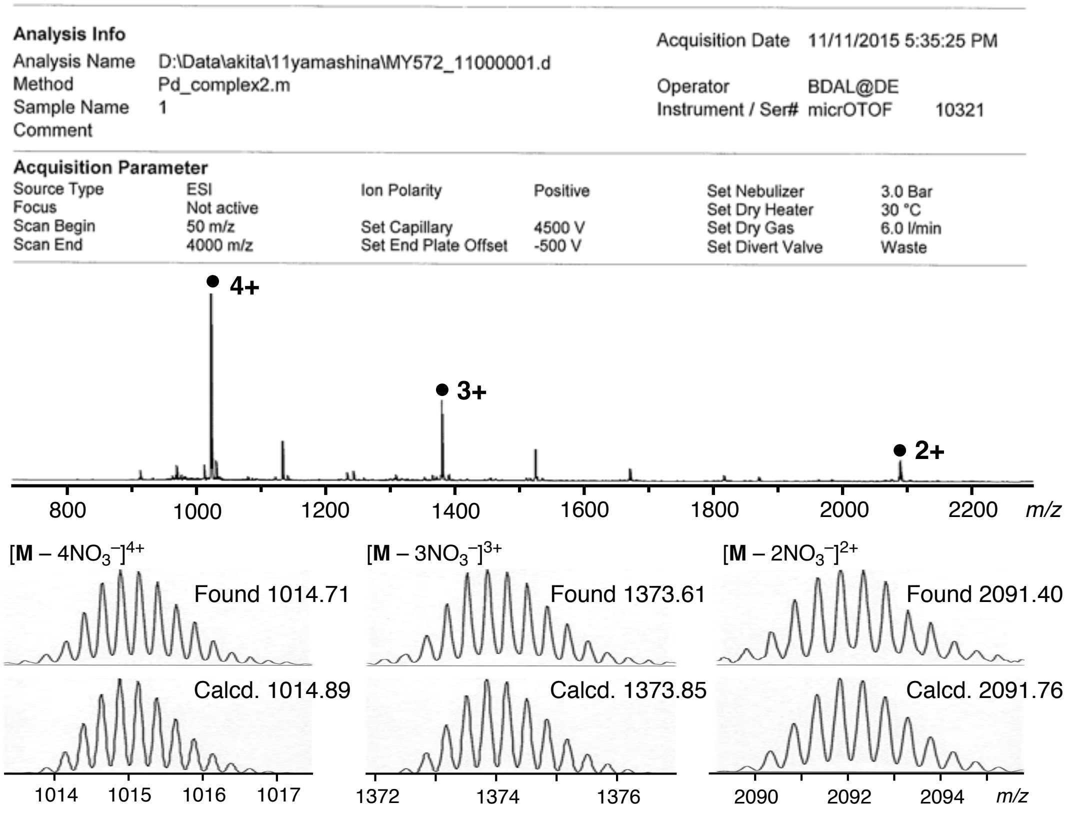

Supplementary Fig. 35 ESI-TOF MS spectrum (H_2O) of **1•9EO**.

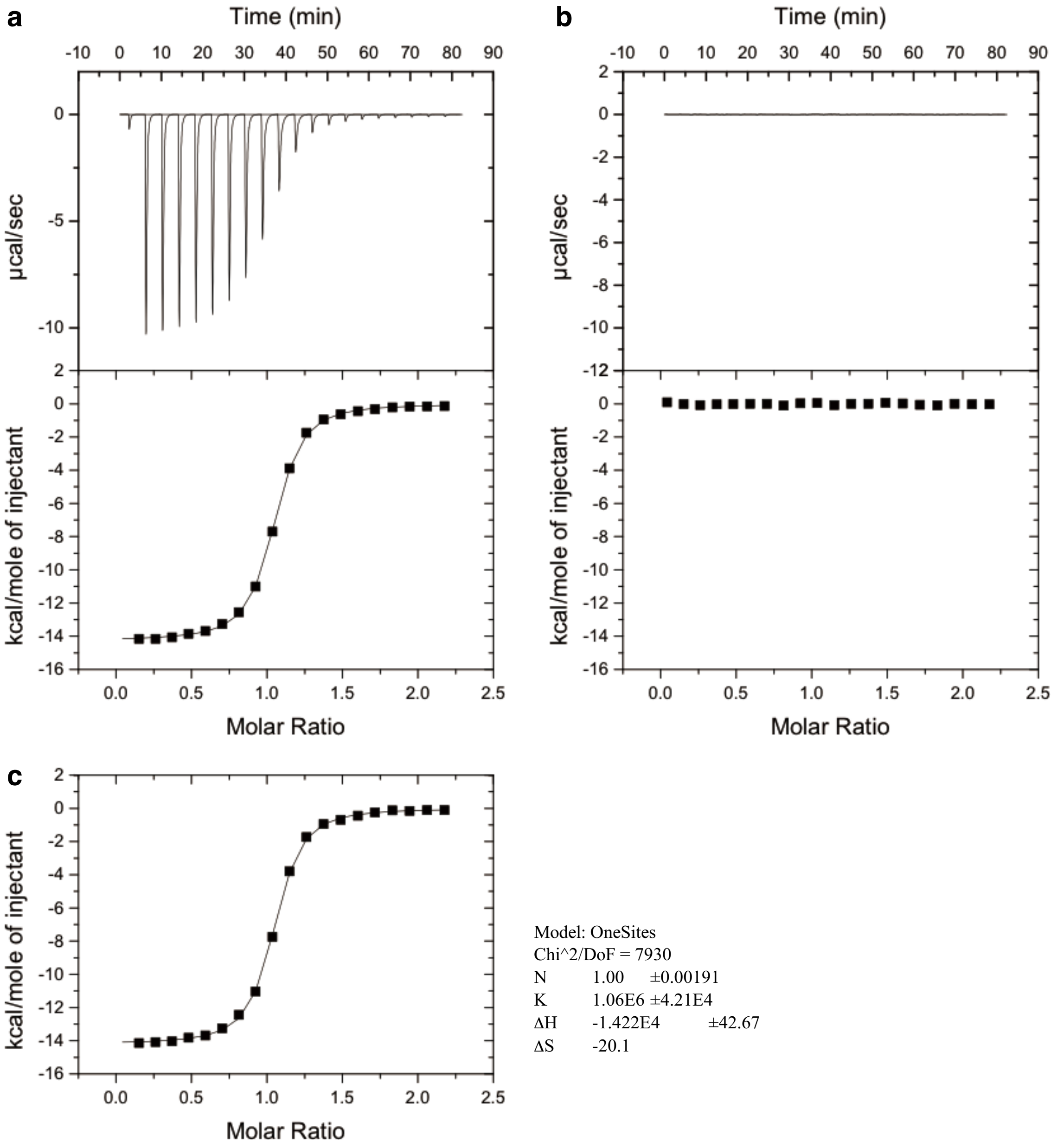

Supplementary Fig. 36 ITC titrations (H_2O, 25 ºC) of **10EO** (3.55 mM) in (a) the presence of **1** (0.11 mM) and (b) the absence of **1**. (c) The solid line represents the best-fitting curve obtained from the Onesites model.

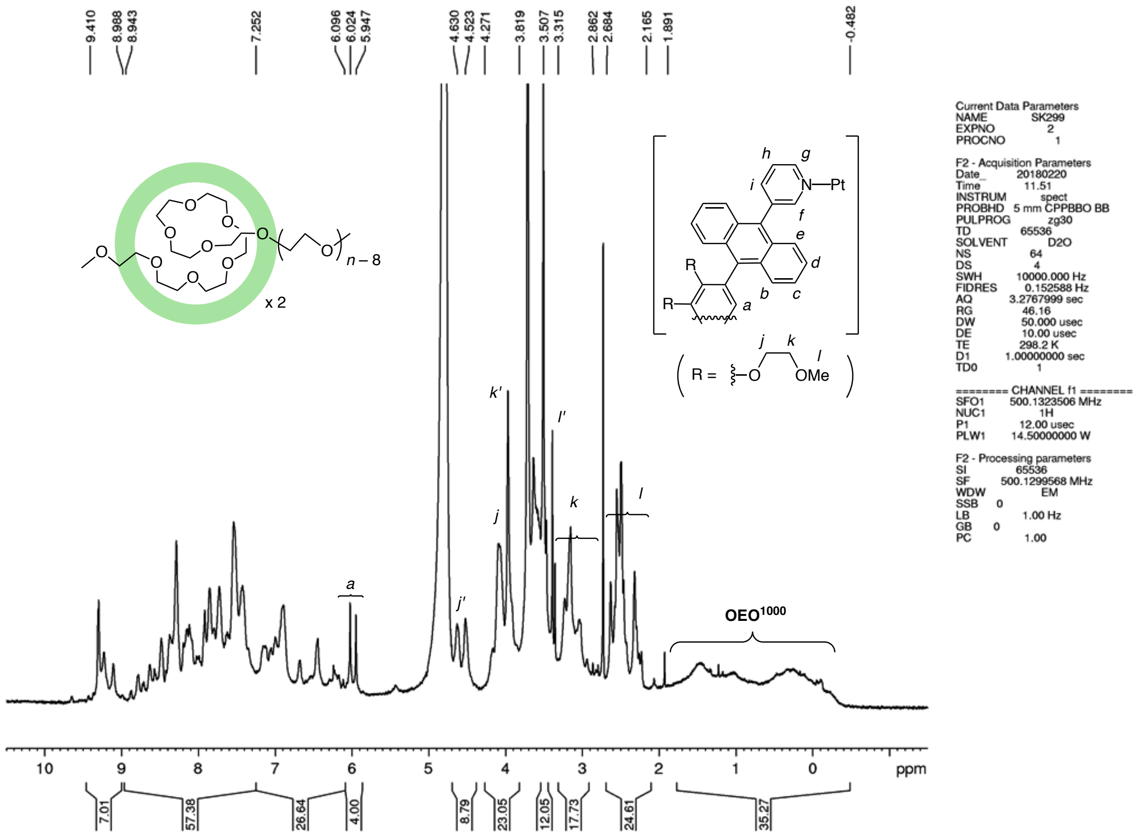

Supplementary Fig. 37 ^{1}H NMR spectrum (500 MHz, D_2O, room temperature) of $(\mathbf{1})_2$•$\mathbf{OEO^{1000}}$.

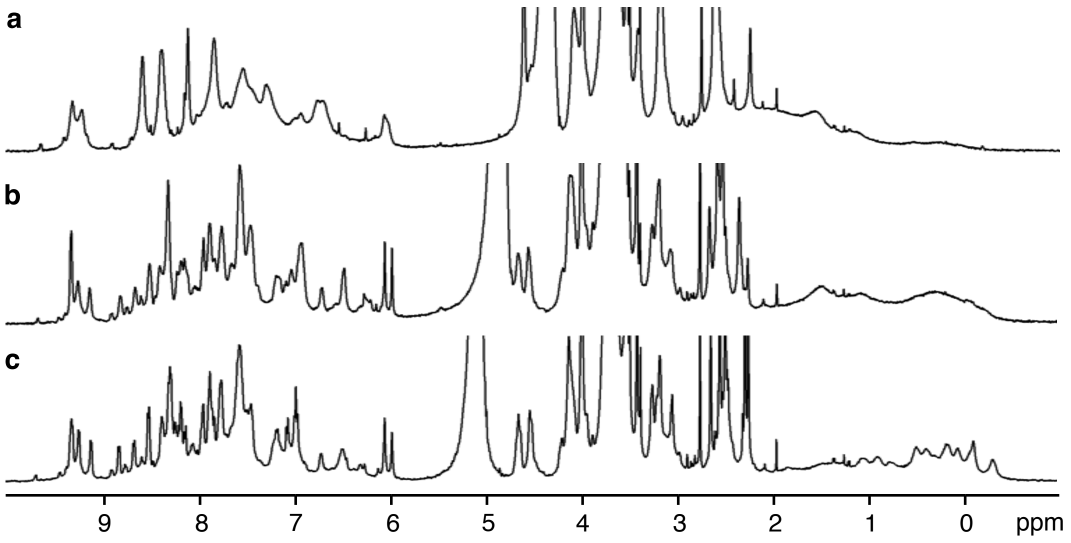

Supplementary Fig. 38 ^{1}H NMR spectra (500 MHz, D_2O, room temperature) of $(\mathbf{1})_2$•$\mathbf{OEO^{1000}}$ at (a) 80 ºC, (b) 25 ºC, and (c) 4 ºC.

Analysis Info Acquisition Date 2018/02/21 18:29:37

Analysis Name D:\Data\akita\16kusaba\SK299\SK299_1\SK299_1000003.d

Method Pd Kusaba01.m Operator BDAL@DE

Sample Name Instrument micrOTOF 213750.10321

Comment

Acquisition Parameter

Source Type	ESI	Ion Polarity	Positive	Set Nebulizer	3.0 Bar
Focus	Not active			Set Dry Heater	30 • ‹C
Scan Begin	50 m/z	Set Capillary	4500 V	Set Dry Gas	6.0 l/min
Scan End	3000 m/z	Set End Plate Offset	-500 V	Set Divert Valve	Waste

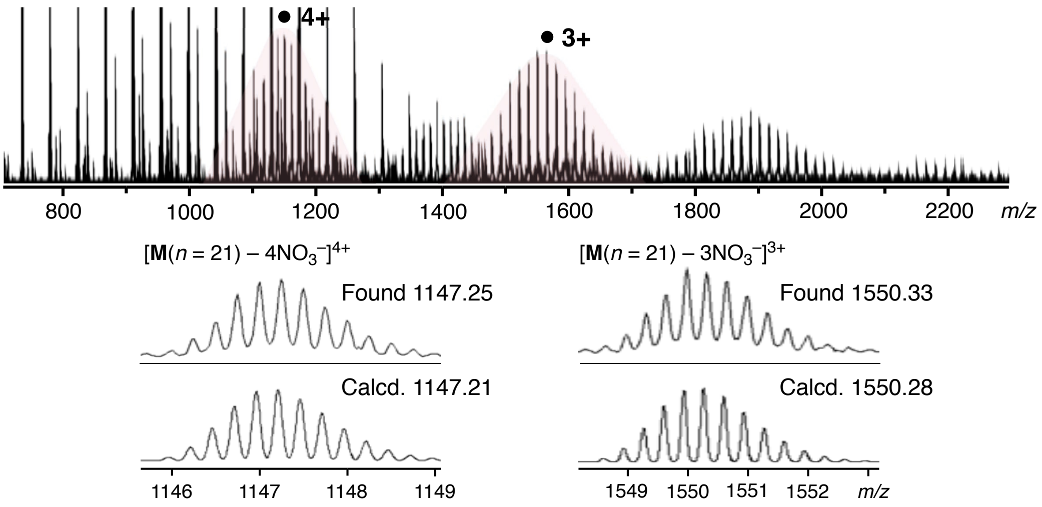

Supplementary Fig. 39 ESI-TOF MS spectrum (H_2O) of $(\mathbf{1})_2$•**OEO**$^{\mathbf{1000}}$.

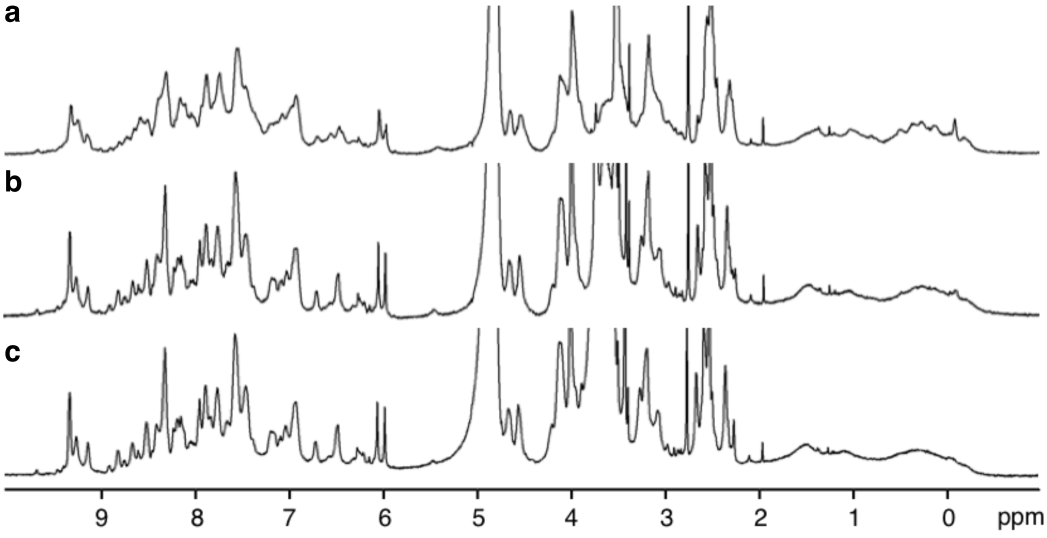

Supplementary Fig. 40 ^{1}H NMR spectra (500 MHz, room temperature) of **1** in D_2O after addition of (a) 0.25, (b) 0.50, and (c) 1.0 equiv. of **OEO**$^{\mathbf{1000}}$.

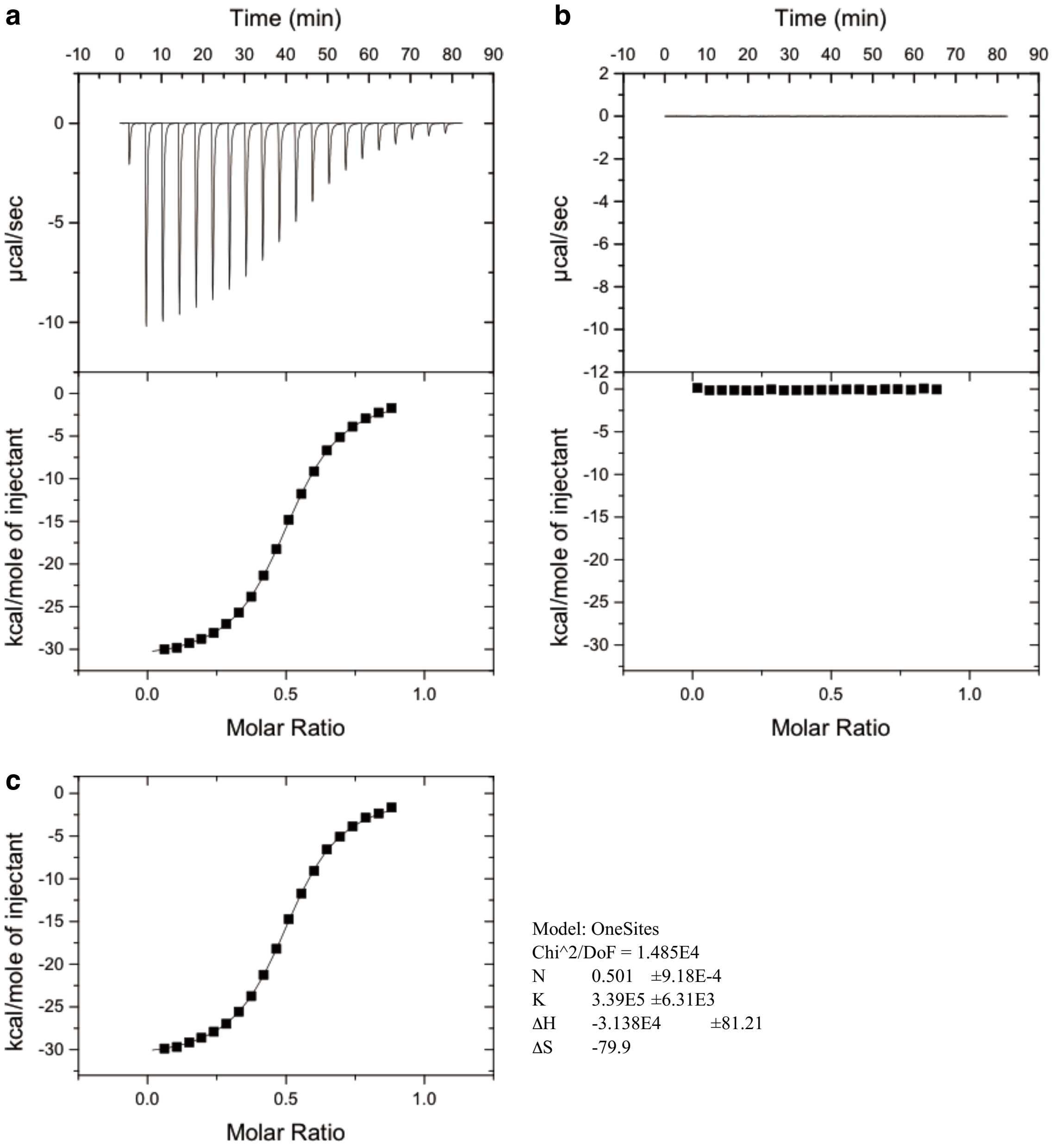

Supplementary Fig. 41 ITC titrations (H_2O, 25 ºC) of **OEO^{1000}** (1.78 mM) in (a) the presence of **1** (0.14 mM) and (b) the absence of **1**. (c) The solid line represents the best-fitting curve obtained from the Onesites model.

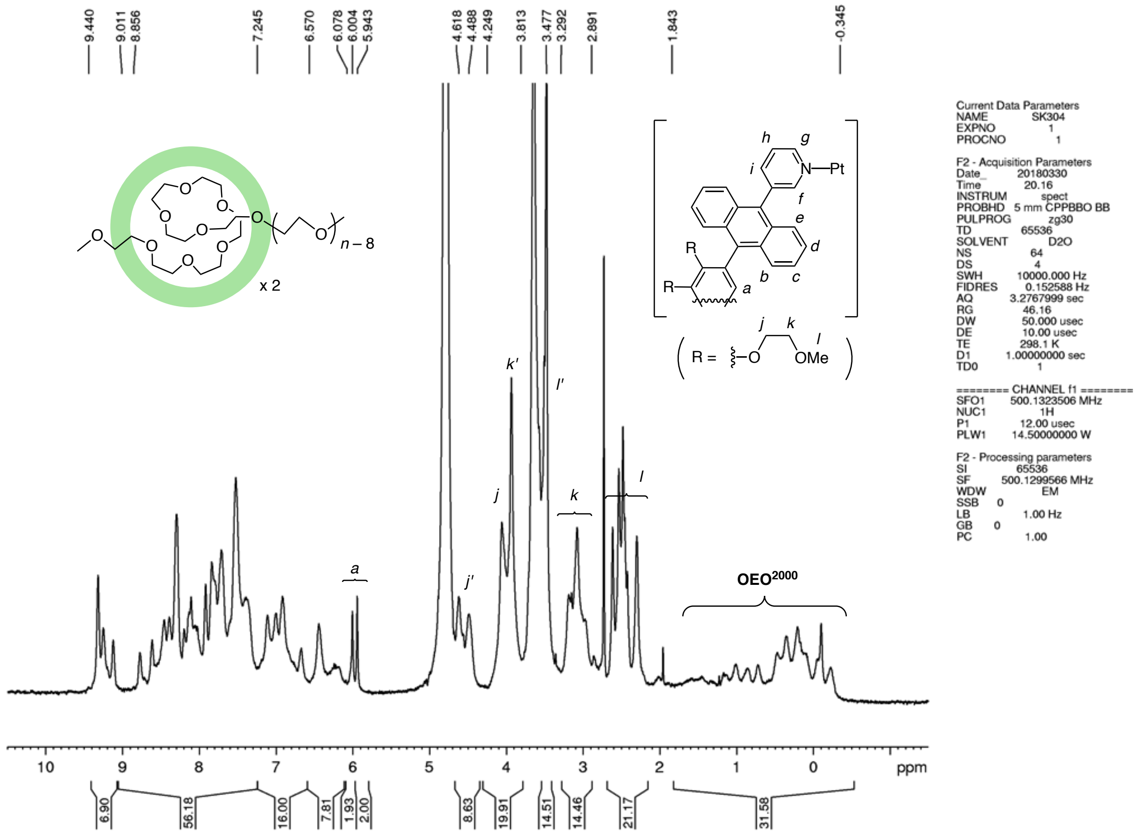

Supplementary Fig. 42 ^{1}H NMR spectrum (500 MHz, D_2O, room temperature) of $(\mathbf{1})_2$•**OEO**$^{\mathbf{2000}}$.

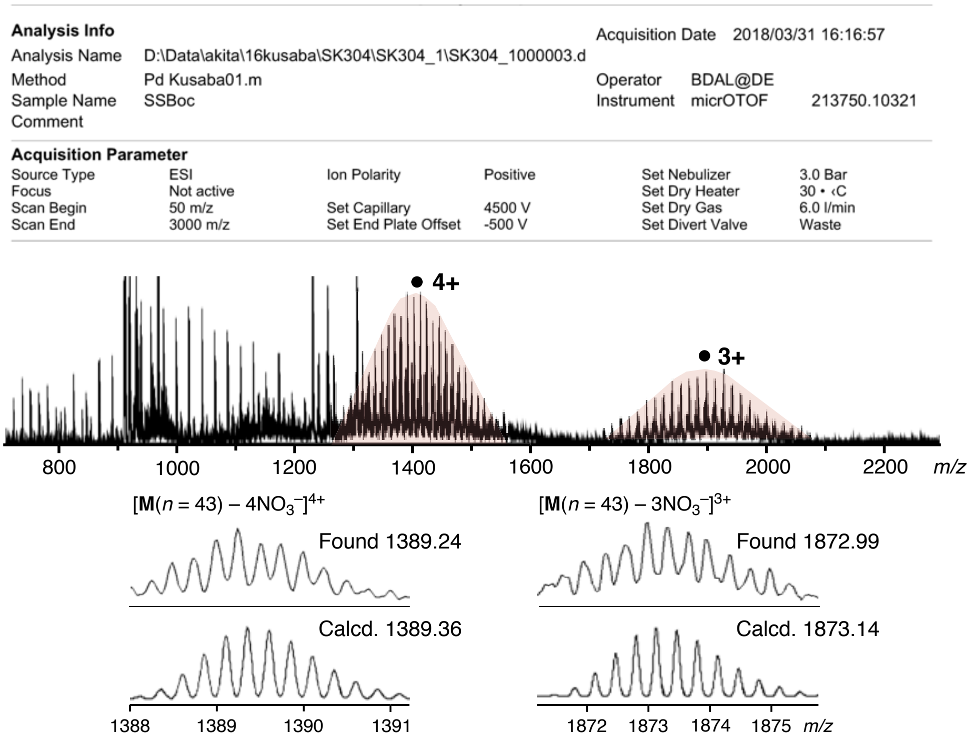

Supplementary Fig. 43 ESI-TOF MS spectrum (H_2O) of $(\mathbf{1})_2$•**OEO**$^{\mathbf{2000}}$.

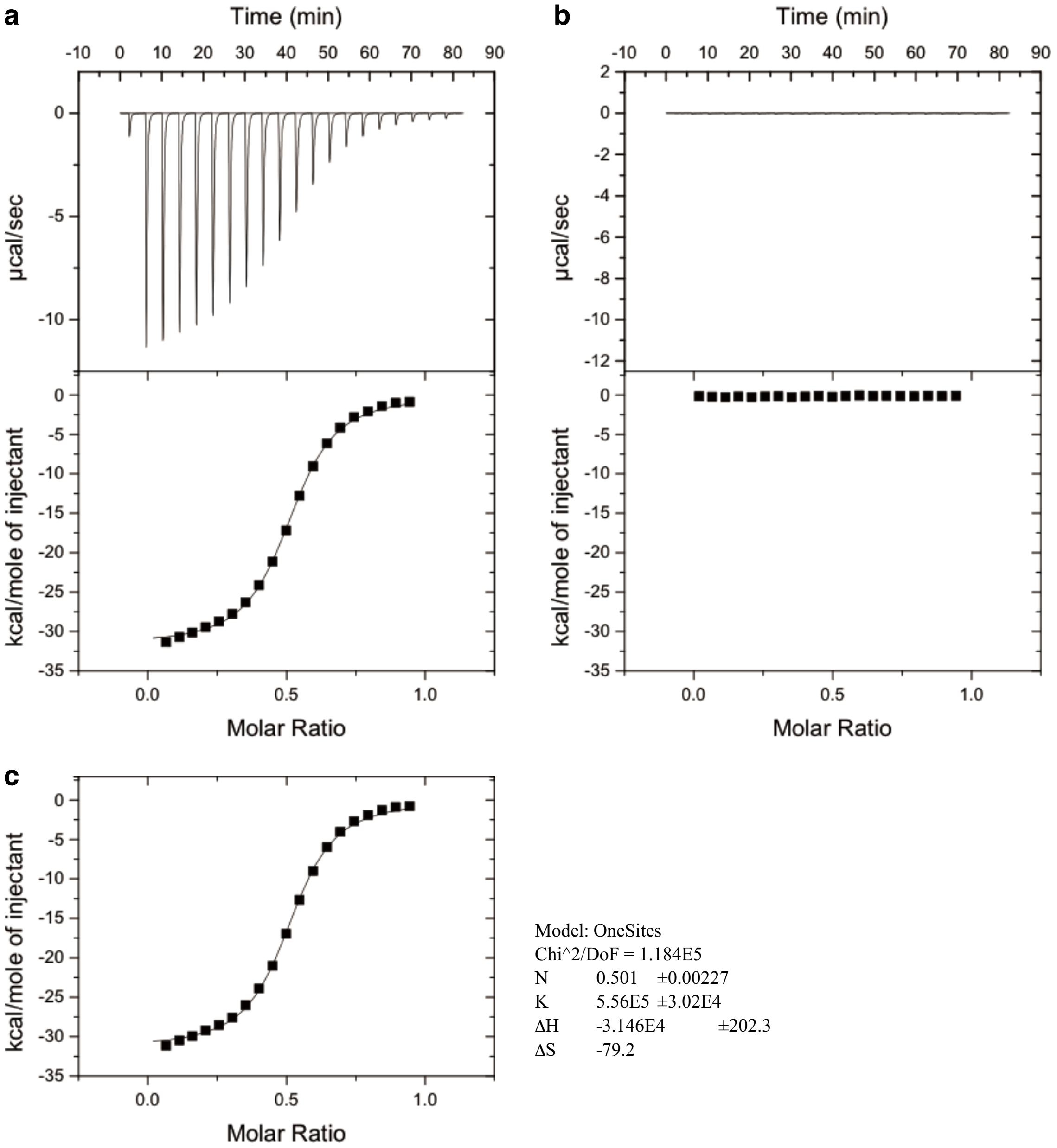

Supplementary Fig. 44 ITC titrations (H_2O, 25 ºC) of **OEO^{2000}** (1.77 mM) in (a) the presence of **1** (0.13 mM) and (b) the absence of **1**. (c) the solid line represents the best-fitting curve obtained from the Onesites model.

Supplementary Table

Supplementary Table 1 Thermodynamic parameters and binding constants (K_a) for the formation of **1•5EO**, **1•CE**, **1•10EO**, (**1**)$_2$•**OEO^{1000}**, and (**1**)$_2$•**OEO^{2000}** obtained by ITC experiments (H_2O, 298 K).

Entry	ΔH [kJ mol^{-1}]	$T\Delta S$ [kJ mol^{-1}]	ΔG [kJ mol^{-1}]	K_a
**1•5EO**	–52.76 ± 0.32	–16.58	–36.18	(2.13 ± 0.22) × 10^6 M^{-1}
**1•CE**	–47.91 ± 0.51	–11.60	–36.31	(2.29 ± 0.43) × 10^6 M^{-1}
**1•10EO**	–59.50 ± 0.18	–25.06	–34.44	(1.06 ± 0.04) × 10^6 M^{-1}
(**1**)$_2$•**OEO^{1000}**	–131.29 ± 0.34	–99.62	–31.67	(3.39 ± 0.06) × 10^5 M^{-2}
(**1**)$_2$•**OEO^{2000}**	–131.63 ± 0.85	–98.75	–32.88	(5.56 ± 0.30) × 10^5 M^{-2}

Supplementary Table 2 Crystal data and structure refinement for **1**.

Identification code	SK287	
Empirical formula	C212 H184 N8 O24 Pt2	
Formula weight	3617.84	
Temperature	93 K	
Wavelength	1.54184 Å	
Crystal system	triclinic	
Space group	P-1	
Unit cell dimensions	a = 21.2847(5) Å	α = 69.915(2) °
	b = 21.4245(5) Å	β = 68.990(2) °
	c = 27.3518(5) Å	γ = 61.486(2) °
Volume	10003.1(4) $Å^3$	
Z	2	
Density (calculated)	1.201 Mg/m^3	
Absorption coefficient	3.092 mm^{-1}	
F(000)	3720.0	
Crystal size	0.065 × 0.054 × 0.041 mm^3	
Theta range for data collection	2.400 to 77.380 °.	
Index ranges	–26<=h<=26, –25<=k<=25, –34<=l<=33	
Reflections collected	80903	
Independent reflections	34070 [R(int) = 0.0393]	
Completeness to theta = 68.13 °	85.3 %	
Absorption correction	multi-scan	
Max. and min. transmission	0.71183 and 1.00000	
Refinement method	Full-matrix least-squares on F^2	
Data / restraints / parameters	34070 / 468 / 2206	
Goodness-of-fit on F^2	1.049	
Final R indices [I>2sigma(I)]	R_1 = 0.0834, wR_2 = 0.2407	
R indices (all data)	R_1 = 0.1248, wR_2 = 0.2714	
Largest diff. peak and hole	3.11 and –1.38 e.$Å^{-3}$	

The supplementary crystallographic data (CCDC 1840603) can be obtained free of charge from the Cambridge Crystallographic Data Centre via www.ccdc.cam.ac.uk/data_request/cif.

Supplementary Table 3 Crystal data and structure refinement for **1•5EO**.

Identification code	SK291	
Empirical formula	C216 H192.66 N8 O25.5 Pt2	
Formula weight	3698.61	
Temperature	93 K	
Wavelength	1.54184 Å	
Crystal system	triclinic	
Space group	P-1	
Unit cell dimensions	a = 19.4231(13) Å	α = 69.368(6) °
	b = 20.4314(14) Å	β = 88.636(5) °
	c = 28.8216(18) Å	γ = 67.662(6) °
Volume	9820.3(12) Å^3	
Z	2	
Density (calculated)	1.251 Mg/m^3	
Absorption coefficient	3.167 mm^{-1}	
F(000)	3809.0	
Crystal size	0.041 × 0.037 × 0.036 mm^3	
Theta range for data collection	2.445 to 50.435 °.	
Index ranges	–19<=h<=19, –18<=k<=18, –26<=l<=28	
Reflections collected	81517	
Independent reflections	20534 [R(int) = 0.1983]	
Completeness to theta = 50.38 °	99.8 %	
Absorption correction	multi-scan	
Max. and min. transmission	0.55274 and 1.00000	
Refinement method	Full-matrix least-squares on F^2	
Data / restraints / parameters	20534 / 2061 / 2161	
Goodness-of-fit on F^2	1.017	
Final R indices [I>2sigma(I)]	R_1 = 0.1273, wR_2 = 0.3098	
R indices (all data)	R_1 = 0.2717, wR_2 = 0.4023	
Largest diff. peak and hole	0.892 and –1.287 e.Å^{-3}	

The supplementary crystallographic data (CCDC 1842338) can be obtained free of charge from the Cambridge Crystallographic Data Centre via www.ccdc.cam.ac.uk/data_request/cif.

Supplementary Methods

Formation of 1·4EO

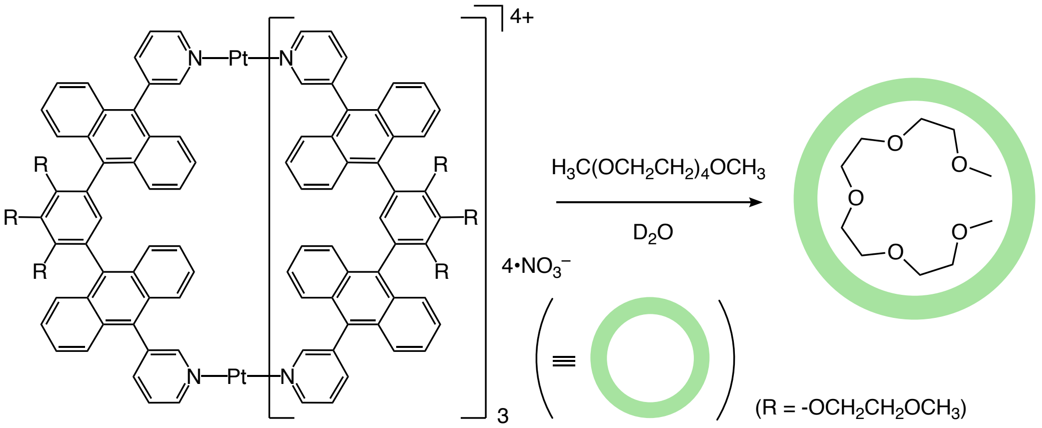

Capsule **1** (1.5 mg, 0.39 μmol), **4EO** (0.10 mg, 0.39 μmol), and D_2O (0.5 mL) were added to a glass test tube. The mixture was stirred at 60 ºC for 30 min. The quantitative formation of **1•4EO** was confirmed by NMR and ESI-TOF MS analyses.

^1H NMR (500 MHz, D_2O, room temperature): δ –0.33 (s, 6H, **4EO**), –0.26-–0.20 (m, 2H, **4EO**), –0.15-–0.03 (m, 4H, **4EO**), –0.01-0.15 (m, 8H, **4EO**), 0.18-0.24 (m, 2H, **4EO**), 2.48 (s, 24H, **1**), 3.11 (m, 16H, **1**), 3.49 (s, 12H, **1**), 3.95 (t, J = 4.0 Hz, 8H, **1**), 3.99 (m, 8H, **1**), 4.09 (m, 8H, **1**), 4.49 (m, 4H, **1**), 4.63 (m, 4H, **1**), 5.96 (s, 4H, **1**), 6.49 (d, J = 9.0 Hz, 8H, **1**), 6.75 (dd, J = 9.0, 7.5 Hz, 8H, **1**), 6.96 (d, J = 9.0 Hz, 8H, **1**), 7.27 (dd, J = 9.0, 7.5 Hz, 8H, **1**), 7.51 (dd, J = 9.0, 7.5 Hz, 8H, **1**), 7.70 (d, J = 9.0 Hz, 8H, **1**), 7.79 (dd, J = 9.0, 7.5 Hz, 8H, **1**), 8.00 (s, 8H, **1**), 8.02 (d, J = 9.0 Hz, 8H, **1**), 8.34 (dd, J = 8.0, 5.5 Hz, 8H, **1**), 8.54 (d, J = 8.0 Hz, 8H, **1**), 9.23 (d, J = 5.5 Hz, 8H, **1**). ESI-TOF MS (H_2O): m/z 1981.7 [**1•4EO** – $2NO_3^-$]$^{2+}$, 1300.5 [**1•4EO** – $3NO_3^-$]$^{3+}$, 959.9 [**1•4EO** – $4NO_3^-$]$^{4+}$.

Formation of 1·6EO

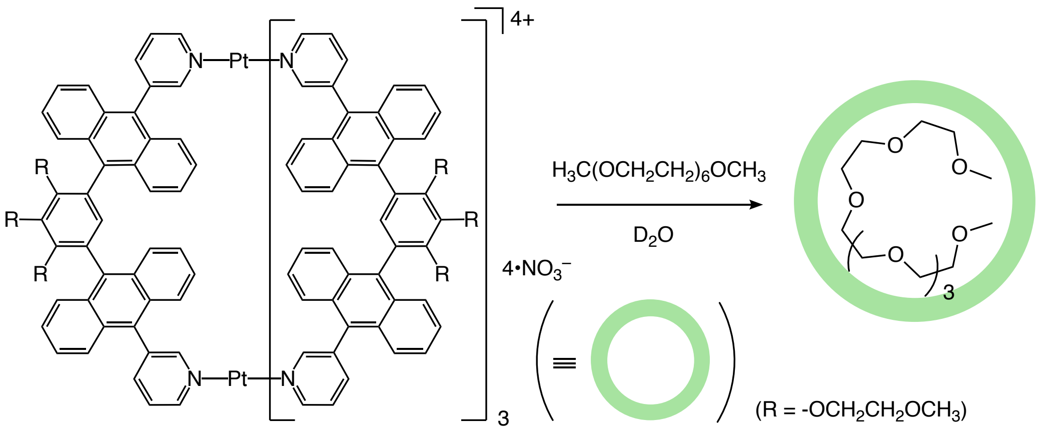

Capsule **1** (1.5 mg, 0.39 μmol), **6EO** (0.10 mg, 0.39 μmol), and D_2O (0.5 mL) were added to a glass test tube. The mixture was stirred at 60 °C for 30 min. The quantitative formation of **1•6EO** was confirmed by NMR and ESI-TOF MS analyses.

^{1}H NMR (500 MHz, D_2O, room temperature): δ –0.30-–0.22 (m, 2H, **6EO**), –0.19 (s, 6H, **6EO**), –0.13-–0.06 (m, 2H, **6EO**), –0.05-0.03 (m, 4H, **6EO**), 0.04-0.19 (m, 8H, **6EO**), 0.20-0.35 (m, 6H, **6EO**), 0.49-0.57 (m, 2H, **6EO**), 2.48 (s, 24H, **1**), 3.11 (m, 16H, **1**), 3.49 (s, 12H, **1**), 3.95 (t, J = 4.0 Hz, 8H, **1**), 3.99 (m, 8H, **1**), 4.09 (m, 8H, **1**), 4.49 (m, 4H, **1**), 4.63 (m, 4H, **1**), 5.95 (s, 4H, **1**), 6.60 (d, J = 9.0 Hz, 8H, **1**), 6.87 (dd, J = 9.0, 7.5 Hz, 8H, **1**), 6.98 (d, J = 9.0 Hz, 8H, **1**), 7.33 (dd, J = 9.0, 7.5 Hz, 8H, **1**), 7.53 (dd, J = 9.0, 7.5 Hz, 8H, **1**), 7.70 (d, J = 9.0 Hz, 8H, **1**), 7.81 (dd, J = 9.0, 7.5 Hz, 8H, **1**), 7.97 (s, 8H, **1**), 8.02 (d, J = 9.0 Hz, 8H, **1**), 8.34 (dd, J = 8.0, 5.5 Hz, 8H, **1**), 8.59 (d, J = 8.0 Hz, 8H, **1**), 9.20 (d, J = 5.5 Hz, 8H, **1**). ESI-TOF MS (H_2O): m/z 2025.8 [**1•6EO** – $2NO_3^-$]$^{2+}$, 1329.8 [**1•6EO** – $3NO_3^-$]$^{3+}$, 981.9 [**1•6EO** – $4NO_3^-$]$^{4+}$.

Formation of 1·7EO

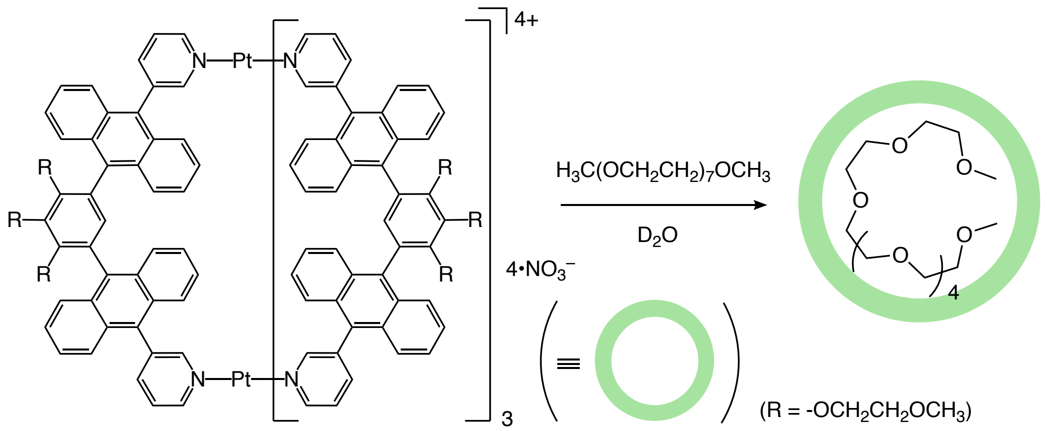

Capsule **1** (1.5 mg, 0.39 μmol), **7EO** (0.10 mg, 0.39 μmol), and D_2O (0.5 mL) were added to a glass test tube. The mixture was stirred at 60 ºC for 30 min. The quantitative formation of **1•7EO** was confirmed by NMR and ESI-TOF MS analyses.

^{1}H NMR (500 MHz, D_2O, room temperature): δ –0.32 (br, 2H, **7EO**), –0.22 (m, 2H, **7EO**), –0.18-–0.03 (m, 10H, **7EO**), 0.03-0.25 (m, 10H, **7EO**), 0.29 (br, 2H, **7EO**), 0.33-0.45 (m, 4H, **7EO**), 0.68-0.79 (m, 4H, **7EO**), 2.51 (s, 24H, **1**), 3.15 (m, 16H, **1**), 3.50 (s, 12H, **1**), 3.96 (t, J = 4.0 Hz, 8H, **1**), 4.04 (m, 8H, **1**), 4.13 (m, 8H, **1**), 4.50 (m, 4H, **1**), 4.62 (m, 4H, **1**), 5.90 (s, 4H, **1**), 6.75 (d, J = 9.0 Hz, 8H, **1**), 6.97 (d, J = 9.0 Hz, 8H, **1**), 7.04 (dd, J = 9.0, 7.5 Hz, 8H, **1**), 7.43 (dd, J = 9.0, 7.5 Hz, 8H, **1**), 7.52 (dd, J = 9.0, 7.5 Hz, 8H, **1**), 7.71 (d, J = 9.0 Hz, 8H, **1**), 7.83 (dd, J = 9.0, 7.5 Hz, 8H, **1**), 7.92 (s, 8H, **1**), 8.00 (d, J = 9.0 Hz, 8H, **1**), 8.35 (dd, J = 8.0, 5.5 Hz, 8H, **1**), 8.65 (d, J = 8.0 Hz, 8H, **1**), 9.14 (d, J = 5.5 Hz, 8H, **1**). ESI-TOF MS (H_2O): m/z 2047.8 [**1•7EO** – $2NO_3^-]^{2+}$, 1344.5 [**1•7EO** – $3NO_3^-]^{3+}$, 992.9 [**1•7EO** – $4NO_3^-]^{4+}$.

Formation of 1•8EO

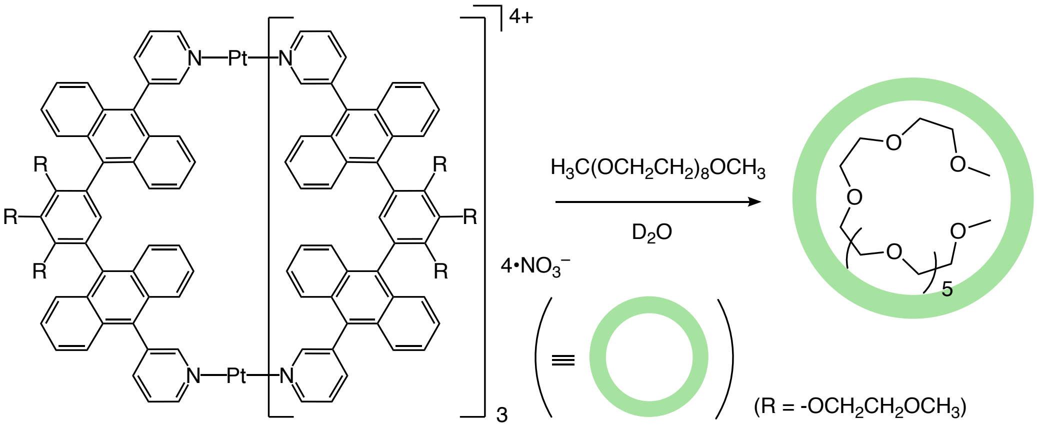

Capsule **1** (1.5 mg, 0.39 μmol), **8EO** (0.20 mg, 0.39 μmol), and D_2O (0.5 mL) were added to a glass test tube. The mixture was stirred at 60 °C for 30 min. The quantitative formation of **1•8EO** was confirmed by NMR and ESI-TOF MS analyses. Under the same conditions, the treatment of **1** with **8EO'** quantitatively afforded **1•8EO'** in water.

1•8EO: ^{1}H NMR (500 MHz, D_2O, room temperature): δ –0.13-–0.04 (m, 8H, **8EO**), 0.08 (m, 2H, **8EO**), 0.12-0.22 (m, 6H, **8EO**), 0.27-0.57 (m, 20H, **8EO**), 0.61 (m, 2H, **8EO**), 2.53 (s, 24H, **1**), 3.18 (m, 16H, **1**), 3.52 (s, 12H, **1**), 3.97 (t, J = 4.0 Hz, 8H, **1**), 4.08 (m, 8H, **1**), 4.14 (m, 8H, **1**), 4.52 (m, 4H, **1**), 4.62 (m, 4H, **1**), 5.87 (s, 4H, **1**), 6.90 (d, J = 9.0 Hz, 8H, **1**), 6.95 (d, J = 9.0 Hz, 8H, **1**), 7.20 (dd, J = 9.0, 7.5 Hz, 8H, **1**), 7.44-7.59 (m, 16H, **1**), 7.72 (d, J = 9.0 Hz, 8H, **1**), 7.85 (dd, J = 9.0, 7.5 Hz, 8H, **1**), 7.88 (s, 8H, **1**), 7.98 (d, J = 9.0 Hz, 8H, **1**), 8.36 (dd, J = 8.0, 5.5 Hz, 8H, **1**), 8.71 (d, J = 8.0 Hz, 8H, **1**), 9.10 (d, J = 5.5 Hz, 8H, **1**). ESI-TOF MS (H_2O): m/z 2069.8 [**1•8EO** – $2NO_3^-]^{2+}$, 1359.2 [**1•8EO** – $3NO_3^-]^{3+}$, 1003.9 [**1•8EO** – $4NO_3^-]^{4+}$.

1•8EO': ^{1}H NMR (500 MHz, D_2O, room temperature): δ –0.39 (br, 2H, **8EO'**), –0.22 (br, 2H, **8EO'**), –0.17-0.15 (m, 16H, **8EO'**), 0.15-0.38 (m, 8H, **8EO'**), 0.67 (br, 2H, **8EO'**), 1.00 (br, 2H, **8EO'**), 2.51 (s, 24H, **1**), 3.15 (m, 16H, **1**), 3.51 (s, 12H, **1**), 3.96 (br, 8H, **1**), 4.06 (m, 8H, **1**), 4.11 (m, 8H, **1**), 4.50 (m, 4H, **1**), 4.62 (m, 4H, **1**), 5.90 (s, 4H, **1**), 6.74 (d, J = 9.0 Hz, 8H, **1**), 6.95 (d, J = 9.0 Hz, 8H, **1**), 7.07 (dd, J = 9.0, 7.5 Hz, 8H, **1**), 7.44 (dd, J = 9.0, 7.5 Hz, 8H, **1**), 7.52 (dd, J = 9.0, 7.5 Hz, 8H, **1**), 7.72 (d, J = 9.0 Hz, 8H, **1**), 7.84 (dd, J = 9.0, 7.5 Hz, 8H, **1**), 8.00 (s, 8H, **1**), 8.00 (d, J = 9.0 Hz, 8H, **1**), 8.35 (dd, J = 7.5, 5.5 Hz, 8H, **1**), 8.65 (d, J = 7.5 Hz, 8H, **1**), 9.14 (d, J = 5.5 Hz, 8H, **1**). ESI-TOF MS (H_2O): m/z 2055.4 [**1•8EO'** – $2NO_3^-]^{2+}$, 1349.6 [**1•8EO'** – $3NO_3^-]^{3+}$,

996.7 [**1•8EO'** – $4NO_3^-$]$^{4+}$.

Competitive binding experiments of *n*EO with 1

A mixture of **5EO** and **4EO** (0.1 mg, 0.39 μmol, each) was added to a D_2O (0.5 mL) of capsule **1** (1.5 mg, 0.39 μmol) in a glass test tube. The mixture was stirred at 60 °C for 30 min. The formation and ratio of **1•5EO** and **1•4EO** were confirmed by ^{1}H NMR analysis. The similar competitive binding experiments revealed the binding preference in the order of **5EO** (volume: 290 Å^3) ≈ **6EO** (340 Å^3) > **7EO** (390 Å^3) > **4EO** (250 Å^3) >> **8EO** (430 Å^3).

Competitive binding experiment of 5EO and CE with 1

A mixture of **5EO** (0.1 mg, 0.39 μmol) and **CE** (0.1 mg, 0.39 μmol) was added to a D_2O (0.5 mL) of capsule **1** (1.5 mg, 0.39 μmol) in a glass test tube. The mixture was stirred at room temperature for 30 min and then at 60 °C for 30 min and 9 h. The formation and ratio of **1•5EO** and **1•CE** were confirmed by ^{1}H NMR analysis.

Formation of 1·9EO

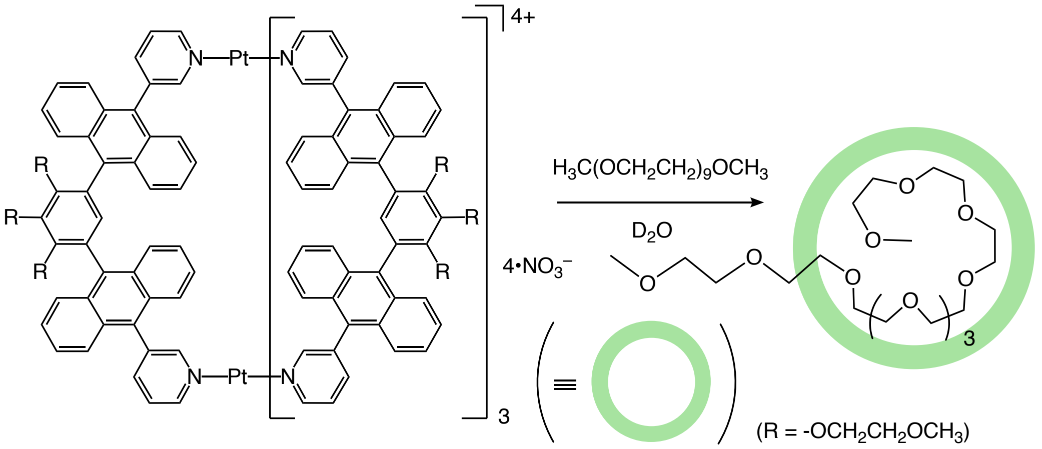

Capsule **1** (1.5 mg, 0.39 μmol), **9EO** (0.20 mg, 0.39 μmol), and D_2O (0.5 mL) were added to a glass test tube. The mixture was stirred at 60 °C for 30 min. The quantitative formation of **1•9EO** was confirmed by NMR and ESI-TOF MS analyses.

^{1}H NMR (500 MHz, D_2O, room temperature): δ 0.00-1.98 (m, 42H, **9EO**), 2.46 (s, 24H, **1**), 3.13 (br, 16H, **1**), 3.48 (s, 12H, **1**), 3.94 (br, 8H, **1**), 4.03 (br, 8H, **1**), 4.08 (br, 8H, **1**), 4.50 (br, 4H, **1**), 4.59 (br, 4H, **1**), 5.94 (s, 4H, **1**), 6.81 (br, 8H, **1**), 6.88-7.10 (br, 16H, **1**), 7.33 (br, 8H, **1**), 7.48 (br, 8H, **1**), 7.67 (br, 8H, **1**), 7.76 (br, 8H, **1**), 7.97 (br, 8H), 8.06 (s,

8H, **1**), 8.29 (dd, J = 7.5, 5.0 Hz, 8H, **1**), 8.61 (d, J = 7.5 Hz, 8H, **1**), 9.17 (d, J = 5.0 Hz, 8H, **1**). ESI-TOF MS (H_2O): m/z 2091.4 [**1•9EO** – $2NO_3^-]^{2+}$, 1373.6 [**1•9EO** – $3NO_3^-]^{3+}$, 1014.7 [**1•9EO** – $4NO_3^-]^{4+}$.

Refinement details of X-ray crystallographic analysis of 1(SK287)

The crystal structure of capsule **1** was solved using SHELXT (Sheldrick, 2015) and then refined with SHELXL (Sheldrick, 2015). Carbon-bound hydrogen atoms were included in idealized positions and refined using a riding model. Disorder atoms were modelled using standard crystallographic methods including constraints, restraints, and rigid bodies. The contribution of the electron density associated with greatly disordered counterions ($4•NO_3^-$) and solvent molecules (H_2O), which could not be modelled with discrete atomic positions, were handled using the solvent mask in the Olex^2 program. There are several error messages (level A and B) in the checkCIF, because of the following reasons. Nevertheless, the quality of the data (R_1 = 0.0834, wR_2 = 0.2407) is more than sufficient to establish the connectivity of capsule **1**.

PLAT029_ALERT_3_A
PROBLEM: _diffrn_measured_fraction_theta_full value Low .   0.855 Why?
RESPONSE: This alert is generated because crystal was severely damaged during the X-ray irradiation. Diffraction intensity in the images of the last part of the schedule was dramatically decayed and the images were not used for the structure refinement.

PLAT413_ALERT_2_A
PROBLEM: Short Inter XH3 .. XHn   H46A   ..H53I   1.88 Ang.
RESPONSE: This alert is generated because there is a large amount of disorder in the structure. In particular, the disordered side chains are very dynamic and may be considered as a solvent. Short contacts between disordered fragments are to be expected.

PLAT220_ALERT_2_B
PROBLEM: Non-Solvent   Resd 1   C   Ueq(max)/Ueq(min) Range   8.2 Ratio
RESPONSE: This alert is generated because there is a large amount of disorder in the structure. In particular, the disordered side chains are very dynamic and may be considered as a solvent, which has considerably large Ueq.

PLAT234_ALERT_4_B
PROBLEM: Large Hirshfeld Difference   O2C   --C47C   0.30 Ang.
PROBLEM: Large Hirshfeld Difference   O6C   --C53C   0.30 Ang.
PROBLEM: Large Hirshfeld Difference   C45B   --C46B   0.28 Ang.
RESPONSE: These alerts are generated because there is a large amount of disorder in the structure. In particular, the disordered side chains are very dynamic and may be considered as a solvent. A disorder model for the side chains could not be built because of the tight

disorder.

PLAT241_ALERT_2_B
PROBLEM: High 'MainMol' Ueq as Compared to Neighbors of O4A Check
PROBLEM: High 'MainMol' Ueq as Compared to Neighbors of C48B Check
RESPONSE: These alerts are generated because there is a large amount of disorder in the structure. In particular, the disordered side chains are very dynamic and may be considered as a solvent. A disorder model for the side chains could not be built because of the tight disorder.

Refinement details of X-ray crystallographic analysis of 1•5EO (SK291)

The crystal structure of host-guest complex **1•5EO** was solved using SHELXT (Sheldrick, 2015) and then refined with SHELXL (Sheldrick, 2015). Carbon-bound hydrogen atoms were included in idealized positions and refined using a riding model. Disorder atoms were modelled using standard crystallographic methods including constraints, restraints, and rigid bodies. The contribution of the electron density associated with greatly disordered counterions ($4•NO_3^-$) and solvent molecules (H_2O), which could not be modelled with discrete atomic positions, were handled using the solvent mask in the Olex^2 program. There are several error messages (level A and B) in the checkCIF, because of the following reasons. Nevertheless, the quality of the data is more than sufficient to establish the connectivity of the capsule and penta(ethylene oxide) structures.

THETM01_ALERT_3_A
PROBLEM: The value of sine(theta_max)/wavelength is less than 0.550
Calculated sin(theta_max)/wavelength = 0.5000
RESPONSE: Refinement was performed using the reflection data of 1.0 Å resolution, since the values of Rint and mean $F^2/\sigma(F^2)$ in the resolution shell between 1.04 and 1.00 Å were 75.5% and 0.23, respectively. Considerably large R values might be due to a poor quality of the crystal and reduced number of parameters used for the refinement. The disordered solvent (water) molecules and nitrate anions could not be modeled and treated with solvent mask of Olex2 program.

PLAT412_ALERT_2_A
PROBLEM: Short Intra XH3 .. XHn H32C ..H50G 1.48 Ang.
PROBLEM: Short Intra XH3 .. XHn H50L ..H52E 1.49 Ang.
RESPONSE: These alerts are generated because there is a large amount of disorder in the structure. In particular, the disordered side-chains are very dynamic and may be considered as a solvent. Short contacts between disordered fragments are to be expected.

RINTA01_ALERT_3_B
PROBLEM: The value of Rint is greater than 0.18

Rint given 0.198

RESPONSE: Considerably large Rint values is due to a poor quality of the crystal and the severely disordered side chains, counterions, and solvents, resulting in weak diffraction at the high angle region.

PLAT020_ALERT_3_B

PROBLEM: The Value of Rint is Greater Than 0.12 .... 0.198 Report

RESPONSE: Considerably large Rint values is due to a poor quality of the crystal and the severely disordered side chains, counterions, and solvents, resulting in weak diffraction at the high angle region.

PLAT026_ALERT_3_B

PROBLEM: Ratio Observed / Unique Reflections (too) Low .. 33% Check

RESPONSE: This alert is generated because there is a large amount of disorder in the structure. This resulted in the weak intensity of the diffraction, where $F^2/\sigma(F^2)$ falls below 2.0 in the resolution shell between 1.36 and 1.26 Å.

PLAT084_ALERT_3_B

PROBLEM: High wR2 Value (i.e. > 0.25) .... 0.40 Report

RESPONSE: Considerably large wR values might be due to a poor quality of the crystal and reduced number of parameters used for the refinement. Refinement was performed using the reflection data of 1.0 Å resolution, since the values of Rint and mean $F^2/\sigma(F^2)$ in the resolution shell between 1.04 and 1.00 Å were 75.5% and 0.23, respectively.

PLAT213_ALERT_2_B

PROBLEM: Atom N1B has ADP max/min Ratio ..... 4.1 prolat

RESPONSE: This alert is generated because there is a large amount of disorder in the structure. A disorder model could not be built because of the tight disorder.

PLAT234_ALERT_4_B

PROBLEM: Large Hirshfeld Difference Pt2 --N2A 0.26 Ang.

PROBLEM: Large Hirshfeld Difference N1C --C1C 0.26 Ang.

PROBLEM: Large Hirshfeld Difference C2C --C3C 0.26 Ang.

PROBLEM: Large Hirshfeld Difference C16D --C17D 0.26 Ang.

PROBLEM: Large Hirshfeld Difference C17C --C18C 0.26 Ang.

PROBLEM: Large Hirshfeld Difference C22B --C23B 0.28 Ang.

PROBLEM: Large Hirshfeld Difference C24B --C26B 0.28 Ang.

PROBLEM: Large Hirshfeld Difference C27B --C32B 0.28 Ang.

PROBLEM: Large Hirshfeld Difference C28B --C29B 0.28 Ang.

PROBLEM: Large Hirshfeld Difference C29B --C30B 0.26 Ang.

PROBLEM: Large Hirshfeld Difference C37B --C38B 0.30 Ang.

RESPONSE: These alerts are generated because there is a large amount of disorder in the structure. In particular, the disordered side chains are very dynamic and may be considered as a solvent. A disorder model for the side chains could not be built because of the tight disorder.

PLAT241_ALERT_2_B

PROBLEM: High 'MainMol' Ueq as Compared to Neighbors of C48C Check
PROBLEM: Low 'MainMol' Ueq as Compared to Neighbors of O4D Check
PROBLEM: Low 'MainMol' Ueq as Compared to Neighbors of C48A Check
RESPONSE: These alerts are generated because there is a large amount of disorder in the structure. In particular, the disordered side chains are very dynamic and may be considered as a solvent. A disorder model for the side chains could not be built because of the tight disorder.

PLAT342_ALERT_3_B
PROBLEM: Low Bond Precision on C-C Bonds .... 0.0416 Ang.
RESPONSE: Considerably large wR values might be due to a poor quality of the crystal and reduced number of parameters used for the refinement. Refinement was performed using the reflection data of 1.0 Å resolution, since the values of Rint and mean $F^2/\sigma(F^2)$ in the resolution shell between 1.04 and 1.00 Å were 75.5% and 0.23, respectively.

PLAT369_ALERT_2_B
PROBLEM: Long C(sp2)-C(sp2) Bond C24B – C26B. 1.59 Ang.
PROBLEM: Long C(sp2)-C(sp2) Bond C39A – C43A. 1.58 Ang.
RESPONSE: These alerts are generated because there is a large amount of disorder in the structure. The positions of concerned carbon atoms are averaged one of disordered structure. A disorder model for the side chains could not be built because of the tight disorder.

PLAT412_ALERT_2_B
PROBLEM: Short Intra XH3 .. XHn H45F .. H47H 1.76 Ang.
RESPONSE: This alert is generated because there is a large amount of disorder in the structure. In particular, the disordered side chains are very dynamic and may be considered as a solvent. Short contacts between disordered fragments are to be expected.
